# Cisplatin exposure alters tRNA-derived small RNAs but does not affect epimutations in *C. elegans*

**DOI:** 10.1186/s12915-023-01767-z

**Published:** 2023-11-29

**Authors:** Manon Fallet, Rachel Wilson, Peter Sarkies

**Affiliations:** 1 Department of Biochemistry, Evolutionary Epigenetics Group, Dorothy Crowfoot Hodgkin Building, University of Oxford, South Parks Rd., Oxford, OX1 3QU UK; 2 Man-Technology-Environment Research Centre (MTM), School of Science and Technology, Örebro University, Fakultetsgatan 1, 70182 Örebro, Sweden; 3 MRC London Institute of Medical Sciences, Du Cane Road, London, W12 0NN UK; 4 Institute of Clinical Sciences, Imperial College London, Du Cane Road, London, W12 0NN UK

**Keywords:** Epigenetics, Small non-coding RNAs, Evolution, DNA damage, Mutation, Epigenetic inheritance, tRNA fragments

## Abstract

**Background:**

The individual lifestyle and environment of an organism can influence its phenotype and potentially the phenotype of its offspring. The different genetic and non-genetic components of the inheritance system and their mutual interactions are key mechanisms to generate inherited phenotypic changes. Epigenetic changes can be transmitted between generations independently from changes in DNA sequence. In *Caenorhabditis elegans*, epigenetic differences, i.e. epimutations, mediated by small non-coding RNAs, particularly 22G-RNAs, as well as chromatin have been identified, and their average persistence is three to five generations. In addition, previous research showed that some epimutations had a longer duration and concerned genes that were enriched for multiple components of xenobiotic response pathways. These results raise the possibility that environmental stresses might change the rate at which epimutations occur, with potential significance for adaptation.

**Results:**

In this work, we explore this question by propagating *C. elegans* lines either in control conditions or in moderate or high doses of cisplatin, which introduces genotoxic stress by damaging DNA. Our results show that cisplatin has a limited effect on global small non-coding RNA epimutations and epimutations in gene expression levels. However, cisplatin exposure leads to increased fluctuations in the levels of small non-coding RNAs derived from tRNA cleavage. We show that changes in tRNA-derived small RNAs may be associated with gene expression changes.

**Conclusions:**

Our work shows that epimutations are not substantially altered by cisplatin exposure but identifies transient changes in tRNA-derived small RNAs as a potential source of variation induced by genotoxic stress.

**Supplementary Information:**

The online version contains supplementary material available at 10.1186/s12915-023-01767-z.

## Background

Environmental factors can influence the phenotype of organisms by interfering with genetic and non-genetic factors [1, 2]. Among non-genetic changes that can be brought about by environmental responses, epigenetics has been defined as changes in the gene expression that are mitotically and/or meiotically inheritable without altering the DNA sequence [3]. Epigenetic alterations known as epimutations, which are transmitted to subsequent generations can be caused by several molecular mechanisms including small non-coding RNAs, DNA methylation, histone modifications, and prions [4–8]. Epimutations may be triggered by environmental stimuli but can also arise spontaneously in the absence of a specific stimulus. In general, the observed epigenetic modifications only remain for a few generations, but they can sometimes last much longer [9–15].

The nematode worm *Caenorhabditis elegans* is an ideal organism to study the scope of epimutations in multigenerational studies thanks to its short generation time. In *C. elegans*, transgenerationally inherited transcriptional silencing is mediated by small RNAs (sRNAs). This silencing is initiated by double-stranded RNA (dsRNA) that will activate RNA-induced epigenetic silencing (RNAe), when the dsRNA is from endogenous origins, or the RNA interference (RNAi) pathway if the dsRNA is from exogenous origins [16]. Both RNA silencing systems have similar machinery: dsRNAs are processed into primary small RNAs (sRNAs) with a length between 18 and 30 nucleotides. Argonaute proteins (AGOs) recognise the sRNAs and form RNA‐induced silencing complexes (RISC). The targeting of transcripts by RISCs is followed by the cleavage of the targeted mRNA and the recruitment of an RNA-dependent RNA polymerase (RdRP) that will recognise the cleaved mRNA and generate secondary sRNAs. These secondary RNAs are antisense to the mRNA target, start with guanine and have a length of 22 nucleotides (22G-RNAs). 22G-RNAs are loaded into a second class of AGOs, the worm-specific Argonautes (WAGOs) that will repress the mRNA target expression at the transcriptional and post-transcriptional level [17]. 22G-RNAs and the associate AGO protein can be transmitted through generations via the gametes resulting in transgenerational persistence of the transcriptional silencing [18]. In addition to epigenetic memory through small non-coding RNA transmission, epigenetic inheritance in *C. elegans* can also be mediated by other epigenetic factors such as changes in chromatin structure. Indeed, the chromatin is highly dynamic, and its organisation regulates gene expression regulation. For instance, in response to a thermal stress, a loss of the histone mark H3K9me3 was associated with a switch towards heterochromatin for specific genomic regions, leading to a long-lasting (14 generations) gene repression [19].

Building on the detailed mechanistic understanding of transgenerational epigenetic inheritance in *C. elegans*, we have recently begun to address the contribution that these processes might make to evolution. Key to these experiments was the use of mutation accumulation lines, which are propagated under minimal selection to enable unbiased assessment of mutations and epimutations regardless of their effect on fitness [20]. These studies demonstrated that epimutations, defined as heritable changes in small non-coding RNAs, chromatin accessibility or gene expression, occur in populations with a rate around 100 times greater than DNA sequence changes. However, epimutations were also ephemeral, lasting only around 4 generations on average [9, 11].

Our previous experiments investigated epimutations in a stable environment. Classical evolutionary theory posits that DNA sequence changes are not affected in an adaptive sense by stress [21]. Whilst the overall rate or type of mutations is clearly affected by certain types of stimuli [22, 23], there is little evidence that the types of genes affected by mutations change such that mutations are more likely to occur in genes involved in stress responses. However, epimutations may behave differently. Our previous work demonstrated that epimutations are more likely to occur in genes involved in environmental responses, even in the absence of stress. Thus, defining how the properties of epimutations are affected by stress is key to understanding what role epimutations may play in evolutionary processes involving adaptation by natural selection.

In this study, we investigated the effect of genotoxic stress on epimutations. We constructed MA lines exposed to the DNA-damaging agent cisplatin, which is already known to induce mutations, and investigated how the rate, spectrum and stability of epimutations were affected. Our results indicated that continuous cisplatin exposure, despite inducing mutations in DNA sequence, had little effect on the occurrence or stability of epimutations, either at the level of gene expression or 22G-RNAs. However, cisplatin exposure led to the induction of heritable changes in fragments of tRNA 3′ halves. Intriguingly, potential target genes showed expression changes associated with tRNA epimutations, suggesting that epimutations in tRNAs might have consequences for gene regulation. Overall, our work suggests that most types of epimutation behave according to the paradigm established for DNA sequence changes, but uncovers tRNA-derived small RNAs, previously implicated in epigenetic processes in mammals but not nematodes, as a new source of transcriptional variability within populations exposed to genotoxic stress.

## Methods

### Construction of mutation accumulation lines

**Fig. 1:**
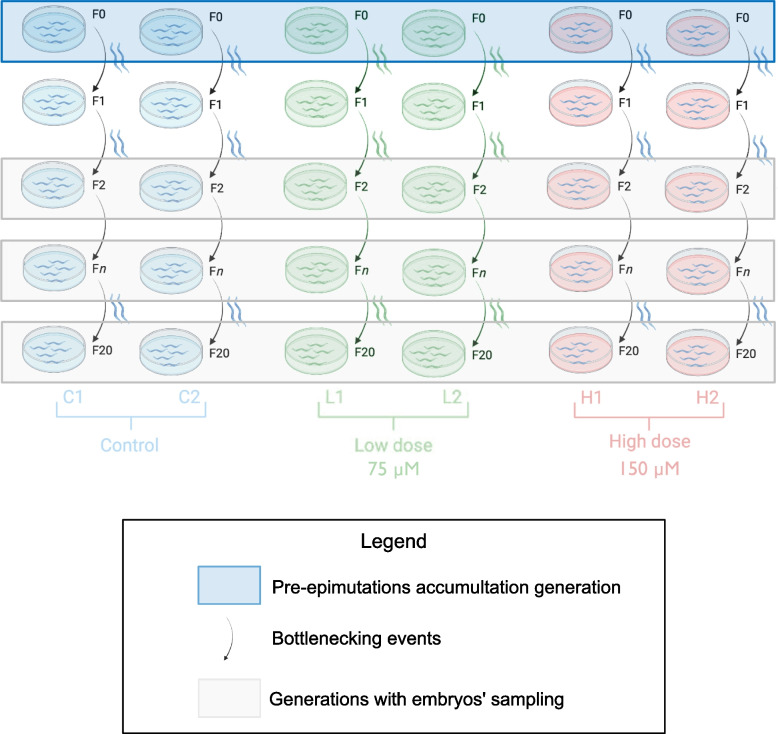
Schematic representation of the experimental design. Six different mutation accumulation lines were constructed. Two lines were maintained in the control condition (blue), two lines were exposed to cisplatin at a low dose of 75 mM (green) and two lines were exposed to a higher dose of 150 mM of cisplatin (red). Each line consisted of a pre-epimutation accumulation generation (F0) that was submitted to 20 successive bottlenecking events for which 2 worms were randomly picked from the previous generation and used as founders of the next generation. Embryos were sampled every two generations for molecular analysis. Figure realised using BioRender and Affinity Designer 2

Mutation accumulation (MA) line experiments in *C. elegans* have been described in previous literature [11, 24]. In brief, MA lines consist of minimising the effective population size by selecting only the minimal number of individuals required to rise each following generation (Fig. 1). According to a previous study [11], two worms were selected to establish each new generation. The *Caenorhabditis elegans* (N2) worms were grown on nematode growth medium (NGM) plates with an OP50 *Escherichia coli* lawn and kept at 20 °C in a temperature-controlled incubator. Six independent lineages were constructed: two lineages in control conditions (lineages A (C1) and B (C2)), two lineages grown on plates enriched with 75 μM of cisplatin (low dose) (lineages C (L1) and D (L2)) and two lineages grown on plates enriched with 150 μM of cisplatin (high dose) (lineages E (H1) and F (H2)). First, N2 worms were bleached to obtain a synchronised population (day 1). On day 2, L1 worms were seeded onto each of the 6 plates (generation 0, pre-accumulation line). On day 4, each of the 6 independent lineages was constructed by picking two N2 L4 hermaphrodite worms from the initial F0 expanded population to a new plate to produce the subsequent generation (generation 2). On day 5, embryos were collected from the original plate (generation 0). This procedure was repeated to propagate the lineages for 20 subsequent generations. Embryos were collected for every two subsequent generations (0, 2, 4, 6, 8, 10, 12, 14, 16, 18 and 20).

### Embryo collection

To collect the embryos, both worms and embryos were washed off plates into 15-ml falcon tubes with 0.1% Triton X. The plates were washed several times to maximise the collection of worms and embryos. The falcon tubes were centrifuged at 2000 RPM for 1 min, and the supernatant was removed. The remaining embryos on the washed plates were loosened from agar by spraying 0.1% Triton X with the pipette angled against the plate. The volume of liquid containing additional washed-off embryos was added to the worm pellet. Three further washes of the pellet were performed until the supernatant was clear to ensure that OP50 bacteria had been removed. The bleaching procedure was used to retain only the embryonic material: the bleaching process destroys the worms whilst leaving the embryos untouched. Worms were bleached using hypochlorite treatment in which 5.5 ml of bleach was added to each sample. Samples were vortexed continuously for 5 min with intermittent checking to ensure worm carcasses were dissolving and to avoid over exposure of embryos to bleach. The bleach solution was washed out rapidly once worm carcasses had disappeared with the addition of M9 to dilute bleach, centrifugation to pellet undissolved embryo material, complete removal of supernatant and repeating this procedure for a total of three washes. Following the 3 post-bleaching washes, the supernatant was removed from the embryo pellet. The pellet was resuspended in 1 ml 0.1% Triton X and then split into 1.5-ml microcentrifuge tubes as follows: 25% of each sample was reserved for RNA library preparation, and 100 μl TRIzol was added to inhibit RNase and maintain RNA integrity. Seventy-five per cent of each sample was reserved for ATAC-seq and frozen with no additive. Microcentrifuge tubes were then submerged in liquid nitrogen before being stored at − 80 °C.

### Extraction of material for assessment of epimutations

#### RNA extraction

RNA was extracted via chloroform isopropanol extraction following standard protocols which had been optimised for our lab [9, 25]. In brief, embryos in TRIzol were lysed through 5 freeze-cracking cycles (frozen in liquid nitrogen and then thawed in a 37 °C water bath). Tubes containing lysed embryos were vortexed for 5 min with 30-s pauses at 30-s intervals and then incubated at room temperature for 5 min for disruption of RNA–protein complexes; 200 μl chloroform per ml of TRIzol was added to each sample followed by vigorous shaking. Samples were then incubated for 2 to 3 min at room temperature followed by centrifugation at a maximum speed at 4 °C for 10 min. The top aqueous layer was aspirated and transferred to a new tube into which 1 μl glycogen, and an equal volume of isopropanol was added. RNA was precipitated overnight at − 20 °C. After overnight precipitation, the samples were centrifuged for 1 h at 4 °C. The supernatant was removed, and 500 μl of 75% ETOH was added to the pellet. The samples were centrifuged at a max speed for 10 min at 4 °C. All ETOH was removed, and the pellet was allowed to air dry. The RNA pellet was resuspended in 10 μl H20. RNA sample concentration and quality were quantified on a 2200 TapeStation instrument using Agilent RNA screen tapes. An RNA Integrity (RIN) score was derived. Samples were additionally quantified using Nanodrop.

#### Small RNA extraction

As previously described [11], the TruSeq Small RNA Prep Kit (Illumina) was used to prepare small RNA libraries from RNA pyrophosphohydrolase (Rpph)-treated RNA. RNA was mixed with 1.5 μl RPPH enzyme and 2 μl 10X NEB Buffer 2 in a final volume of 20 μl. This was incubated at 37 °C for 1 h. The number of PCR cycles was increased from 11 to 15 as per protocol optimised previously [9]; otherwise, all steps were done according to the manufacturer’s instructions. Size selection was performed using gel extraction with a 6% TBE gel (Invitrogen). Validation of 22G-RNA libraries was done on a 2200 TapeStation (Agilent).

#### Assay for transposase accessible chromatin (ATAC)

The protocol for ATAC was adapted from Daugherty et al. [26] after the original method for ATAC-seq [27] and was previously described [11]. In brief, the nuclei were extracted from embryo samples through three cycles of freeze cracking; 200 μl of nuclear preparation buffer was added to each microcentrifuge tube containing frozen embryos, sample submersion in liquid nitrogen for 90 s and then transferred to a 37 °C water bath for 90 s. Embryonic material was then transferred to a Wheaton glass tissue homogeniser in ice and was ground (3.5 grinds), and vessels containing the embryonic material were covered and centrifuged for 2 min at 200 RCF at 4 °C. The supernatant containing the nuclei was transferred to a microcentrifuge tube on ice. The remaining embryo material was ground again, spun down and the supernatant again transferred to the collection tube. This cycle was repeated 4 times. Collection tubes were spun at 1000 RCF at 4 °C for 10 min, and the supernatant was discarded, leaving the nuclei pellet behind. To each sample nuclei pellet, tagmentation enzyme and tagmentation buffer were added (Illumina Tagment DNA enzyme and buffer). The samples were incubated for 30 min at 37 °C on a thermoshaker set to 580 RPM. Samples then underwent DNA clean up with a Qiagen MiniElute Reaction Cleanup Kit. The resulting DNA was eluted in 10 μl of EB buffer. PCR adapters from the Illumina Nextera DNA prep kit (Illumina) were selected and added to each sample along with PCR master mix. PCR was run with the following cycle parameters: 72 °C for 5 min, 98 °C for 30 s, 14 cycles (98 °C for 10 s, 63 °C for 30 s, 72 °C for 1 min), 4 °C hold. Size selection isolates the desired DNA fragments and was achieved with magnetic AMP X beads (Beckman Coulter). First, excessively large DNA fragments were removed as follows: 25 μl AMP X bead solution was added to each sample. Samples were incubated at room temperature for 10 min. The PCR tubes containing the bead and sample mix were then put onto a magnetic rack for 5 min until the sample appeared clear. The samples were transferred without disturbing the magnetic beads to a new set of PCR tubes. To remove excessively small DNA fragments, the same procedure was followed, but the volume of AMP X bead solution added was adjusted to 65 μl. After incubating on the magnetic rack, the clear liquid was carefully removed and discarded. At this point, the beads had the desired library bound. The bead pellet was washed twice with 80% EtOH and then allowed to air dry at room temperature for 2 min. Excess EtOH droplets were removed with a pipette tip from inside each PCR tube; 22 μl of nuclease-free water was added to each sample, and the beads were washed down and dispersed into the liquid. The PCR tubes were returned to the magnetic rack, the DNA having eluted into the nuclease-free water. The liquid was removed without disturbing the magnetic beads to a final collection tube which could then be frozen at − 80 °C. Validation of ATAC libraries was performed by quantification on the 2200 TapeStation using Agilent D1000 Screen tapes. ATAC libraries were also quantified on Qubit using the Qubit dsDNA HS Assay kit from Invitrogen.

### Library construction

#### RNA library construction

cDNA library preparation and PolyA-selected RNA sequencing were carried out in the MRC LMS Genomics Facility to generate PE100bp reads. A cDNA library from 31 samples was prepared using the TruSeq RNA Sample Prep kit (Illumina) following the manufacturer’s instructions. The cDNA library was then hybridised to the flow cell of the Illumina HiSeq 2500 and sequenced. The library was run on three separate lanes to achieve good coverage depth. The sequencing data were processed by the instrument’s Real Time Analysis (RTA) software application, version 1.18.64. De-multiplexing was done within the sequencing facility with CASAVA.

#### ATAC library construction

For submission to the LMS Genomics Facility, multiplexing of samples was performed, with 10–12 samples grouped into a pool. Samples were combined as 10-nM dilutions. Paired-end sequencing of ATAC samples was done in the LMS Genomics Facility on a HiSeq instrument. The sequencing data were processed by the instrument’s Real Time Analysis (RTA) software application, version 1.18.66.3, using default filter and quality settings. Demultiplexing was done within the sequencing facility using CASAVA- 2.17, allowing zero mismatches.

#### Small RNA library construction

For submission to the LMS Genomics Facility, 3-nM dilutions were prepared from each small RNA sample; 3-nM sample dilutions were pooled at ~ 30 samples per pool with unique indexes. Sample pools were validated on TapeStation and quantified on Qubit using dsDNA reagents. MiSEQ single-read sequencing (50-bp read length) was used to validate sample pools. Balancing or preparation of new pools was undertaken to improve the balance of samples in the pool as needed. Pools with a variation in total read count between samples greater than 20% were balanced on the basis of the MiSEQ read count estimation. High-output single-read sequencing was performed on the NExtSeq 2000 instrument in the LMS Genomics Facility. The max read length was 60 bpm. The sequencing data were processed by the instrument’s Real Time Analysis (RTA) software application, version 2.11.3.

### Pre-processing and alignments

#### RNA-seq libraries

RNA fastq files were aligned to the *C. elegans* genome using Bowtie2 [28] to produce a file containing the raw counts data. Sam files were sorted and converted to bam using samtools [29] and then bed files using bedtools [30]. Sorted counts were intersected with coordinates of coding genes derived from the UCSC genome browser [31] using BEDTools intersect commands [30].

The files were simplified so that only the longest transcript was represented (the other isoforms were filtered out) and 21U-RNAs were removed. In addition, all genes for which no expression was seen in any of the samples were removed. The read values were normalised using DESeq2 [32]. DESeq2 is using the median of ratios method to normalise data. In brief, a pseudo-reference sample was created for each gene using the geometric mean of all samples. Then, the ratio sample/pseudo-reference sample was calculated for every gene in a sample. The median of these ratios was subsequently calculated to obtain the normalisation factor for each sample. Finally, normalised count values were obtained by dividing each raw count value of a given sample by the sample’s normalisation factor.

#### Small RNA

Secondary processing and demultiplexing of data were done within the sequencing facility with Bcl2fastq 2_2.20.0 (Illumina). Identification of the small RNAs was further done using the tinyRNA pipeline (tinyRNA-1.0.1) [33] following the standard parameters. Only the following options have been adapted: adapter sequence to trim = AGATCGGAAGAGCACACGTCTGAACTCCAGTCAC; Trim bases from the tail of a read = 15. Read counts were then normalised using DESeq2 [32].

#### ATAC-seq libraries

Trimming of reads to remove adapters was performed using the FASTX Toolkit from the Hannon Lab (RRID:SCR_005534). Sequence alignment was carried out using Bowtie2 [28] to produce a file containing the raw count data. Sam files were sorted and converted to bam using samtools [29].

### Identification of gene expression changes

Global differentially expressed genes (DEGs) using the two lines per condition and the generations as replicates were identified with DESeq2 [32] by comparing low-dose and control conditions and high-dose and control conditions. Significant gene expression changes were identified on the basis of an adjusted *p*-value < 0.05.

For the detection of specific gene expression changes observed in each generation, we used the DESeq2 normalised count matrix. Then, gene expression changes were identified using a local regression model (loess) in which log2(mean counts) for each generation (1 to 20) in each line (C1, C2, L1, L2, H1 and H2) were plotted against the log2(mean counts) of the generation 0 (pre-epimutations accumulation generation) for each gene in line with the approach taken in previous work to identify changes in RNAs [9]. For each gene, in each generation, within each line, a *Z*-score was derived by subtracting the mean of the residuals from the linear model from each individual residual value and dividing the output by the standard deviation of the residuals. To detect significant epimutations (significant gene expression deviation from the ancestral line), a *Z*-score cut-off was used. This cut-off threshold was previously calculated in previous work [11]. Briefly, the *Z*-score threshold was defined by comparing, across all *Z*-scores, the number of epimutations observed in two or more consecutive generations in a real dataset and in a simulated one. This number was significantly greater in the real dataset when the *Z*-score was at a maximum of 2.25 (Additional file 1: Fig. S1A). Therefore, genes with a *Z*-score greater or less than 2.25 were defined as showing differential accessibility. Genes with a *Z*-score greater than 2.25 were annotated as “up” epimutations and regions with a *Z*-score less than 2.25 were annotated as “down” epimutations (Additional file 1: Fig. S1B). The *Z*-scores obtained for expression changes and epimutations were cut-offs and used to get binarised tables (*Z*-scores <  − 2.25 set up as “ − 1” (down epimutation); − 2.25 < *Z*-score < 2.25 as “0” and *Z*-scores > 2.25 as “1” (up epimutation)) for each locus as previously described [11].

### Identification of genetic mutations

ATAC-seq data were used to identify SNPs and indels. Briefly, the *C. elegans* genome (version WS252) was indexed using samtools [29] and the following command: *samtools index genome.fa*. Bam files from the ATAC-seq libraries preparation were used to generate pileup files using samtools [29], and VarScan (v2.4.5) [34] was used to detect DNA mutations in each sample.

In each lineage, DNA mutations detected in F0 were used as a baseline and discarded from each subsequent generational dataset. In mutation accumulation lines, we aim to look only at the mutations that got fixated. Then, true and consistent mutations were separated from the others by keeping only the mutations that appeared in each of the generations and remained until the last generation. We kept data only for the generations that could be verified using the datasets of the following generations. Therefore, the new DNA mutations appearing only in the last generations available (generation 20 for control lineages, 16 for LD lineages, and 18 for HD lineages) were discarded from the analysis. Fixated DNA mutations were identified in each generation and each lineages using a customised R script. Tables were computed for SNPs and indels with the positions of the mutations for each generation. A gene was considered affected by a DNA mutation when one was found within the gene body including exons and introns. Sum-up tables were then manually constructed with the total number of genes affected by DNA mutations, the generation to which the epimutation appears, the nucleotide of reference and the new one after SNP. Reference codons and new ones after indels were recorded using the jbrowser (Wormbase) and the input from Varscan.

### Identification of small RNA epimutations

The feature counts table computed by the tinyRNA pipeline was used as input for subsequent analysis on Rstudio (R version 4.2.1 [35]). Data from all sRNAs were later sorted by type of interest: 22G-RNAs, 26G-RNAs, piRNAs and miRNAs using the value or tag identified by the tinyRNA pipeline (Additional file 2: Table S1). tRNAs were sorted among the unknown (“unk” in Additional file 2: Table S1) reads from the tinyRNA pipeline using the reference annotation WS279 (WormBase Release WS279_master.gff3). Epimutations were identified using a linear model in which log2(mean counts) for each generation (1 to 20) were plotted against the log_2_(mean counts) of the generation 0 (pre-accumulation line) for each gene in line with the approach taken in previous work to identify epimutations in small RNAs [9]. For 22G-RNAs, the total number of 22G-RNAs mapping antisense to each protein-coding gene was used as the input for a linear model comparing each generation in each lineage to the parent generation for that lineage. The *Z*-score of the residuals from the linear model was extracted. Genes with an absolute *Z*-score ≥ 2.25 were selected as putative epimutations, in line with the cut-off used for gene expression analysis (see above) and used in our previous study [11]. For piRNAs, 26G-RNAs, tRNAs and miRNAs, the number of reads matching exactly to the annotated loci was used as the input for the linear model, and putative epimutations were identified as for total 22G counts for genes.

### Test for gene expression changes/epimutation inheritance

All our datasets are derived from a pre-mutation founder generation and comes from alternate embryo sampling realised every two generations (2, 4, 6, 8, 10, 12, 14, 16, 18 and 20). We had to make certain assumptions about the epimutation status for the missing generations and considered for instance that an epimutation appearing in generation 2 and present in generation 4 would exist in generation 3 (even if data were not collected at that generation). Thus, in order to be considered to be inherited, we required that two consecutive even-numbered generations displayed an epimutation. Moreover, due to insufficient yield of RNA or cDNA, some samples were omitted from sequencing (RNA-seq dataset generation 12 for the line C2 and generation 20 for the line H1; small RNA-seq dataset generation 6 for the lines C2, L2 and H1; generation 10 for the line L1; ATAC-seq dataset generations 8, 10 and 14 for the line C2; generations 6, 14, 18 and 20 for the line L1; generations 2, 6, 14,18 and 20 for the line L2; generations 12, 14, 16 and 20 for the line H1; and generations 6 and 20 for the line H2).

In addition, each change of directionality in an epimutation from a generation to the subsequent one (for instance “up” epimutation at generation 2 and “down” at generation 4) was considered to be a termination of the preceding epimutation and commencement of a new epimutation run.

### Characterisation of tRNA fragments in epimutation accumulation lines

For further characterisation, *C. elegans* tRNAs were downloaded from http://gtrnadb.ucsc.edu. The sequences were then aligned to the genome using Bowtie [28] with zero mismatches. The resulting sam file was converted to bam using samtools [29] and bed using bedtools [30] to give coordinates of tRNA genes. To assign short non-coding RNAs to tRNAs, alignment files for 18–36 nucleotide small RNAs generated by tinyRNA (above) were converted to bed files using bedtools and then intersected with the tRNA coordinates using bedtools intersect. The position of the start of the read relative to the tRNA sequence was obtained from this file using a custom script in R. Readcount for each small RNA mapping to a tRNA was normalised using the size factors from DESeq calculated as part of the tinyRNA pipeline. Counts for individual tRNA types were obtained by summing normalised reads mapping to the 3′ half of each tRNA type and this table (Additional file 3: Table S2) was subsequently used for further analysis to identify epimutations in tRNAs according to the procedures in the “Identification of small RNA epimutations” section. To identify potential target genes, small RNAs corresponding to tRNA 3′ halves were extracted from all the lines and combined into a single file, along with the annotation indicating which tRNA the sequence was derived from. This was aligned to the cell genome allowing up to three mismatches. Protein-coding genes overlapping with mismatched tRNA fragments were then obtained using bedtools intersect, to identify genes that were potentially targeted by tRNAs (Additional file 4: Table S3). Code for this analysis and the figures is on the GitHub page.

### Identification of Argonautes that bind tRNAs

Fastq files corresponding to sequences immunoprecipitated in association with *C. elegans* Argonautes were downloaded from the SRA via GSE208702 [18]. Fastq files were converted to fasta files and aligned to cell using bowtie [28] with zero mismatches and recording only one match per sequence. The resulting sam files were converted to bam using samtools [29] and bed files using bedtools [30]. These files were intersected with the tRNA annotations as above, and reads greater than 25 nucleotides were selected to ensure that no overlapping 22G-RNAs were included. Reads were normalised to the total read count and further normalised by the paired input for each IP. Total tRNA normalised tRNA counts were then visualised to identify which AGOs appeared to show notable binding. To test whether the identified Argonautes were required to stabilise tRNAs, small RNA reads were downloaded from mutants lacking ergo-1, wago-10 and rde-1 as well as N2 (WT) [18], and reads were assigned to tRNA fragments as before, again ensuring that 22G-RNAs were not included. Reads were normalised to total read count. Total tRNA counts were compared for the mutants. The apparent decrease in wago-10 mutants was then explored further by investigating counts mapping to different tRNA types across the three replicates, using a Wilcoxon unpaired test to identify any that showed significant differences between N2 and wago10 mutants. All code for the R analysis including figure plotting commands is on the GitHub page.

### Integration of gene expression change and epimutations data

To match up gene expression changes and sRNA epimutations, we integrated data in a gene-centric manner. For each gene, we recorded its expression state, if expression state change was inherited and the presence of epimutated sRNAs. We also recorded the directionality of each change (up or down gene expression change or epimutation) and the generations affected by both the gene expression change and the epimutation to investigate simultaneous (at the same time) and concordant (in the same direction) changes.

### Gene ontology analysis

We computed lists of genes with expression changes or epimutation and uploaded them to WormEnrichr [36, 37]. The number of genes annotated for each identified ontology term was compared between the test lists and the background sets of genes (with no epimutations or expression changes). We set up as a requirement a minimum of five genes to be present in either the test or background sample gene list for the ontology term to be included in the analysis. Fisher’s exact test was used to calculate the enrichment of the test list of genes for ontology terms in comparison to the background lists.

### Specific investigation of epimutation occurrence in apoptotic genes

As cisplatin is inducing DNA damage and oxidative stress resulting in cell death [38], we specifically looked at epimutations occurring in apoptotic genes in *C. elegans*. For that purpose, we downloaded from Wormbase (https://wormbase.org/) the list of the 117 genes involved in apoptosis (Additional file 5: Table S4). The rate of new epimutations arising in these genes, for both gene expression and tRNA 3′ halves epimutations, was calculated as described above. Differences in epimutations rate between conditions were tested using the Kruskal–Wallis rank sum test. The difference was considered significant when *p*-value < 0.05.

### Production of plots and figures

All plots and statistical analysis were made using R [35] and RStudio: RStudio Team (2022). RStudio: Integrated Development Environment for R. RStudio, PBC, Boston, MA, URL: http://www.rstudio.com/. All code for the R analyses including figures plotting commands is available on the GitHub page.

Plots and figures were then processed with Affinity Designer 2.

## Results

### Chronic cisplatin exposure induces genotoxic stress and gene expression changes

**Fig. 2:**
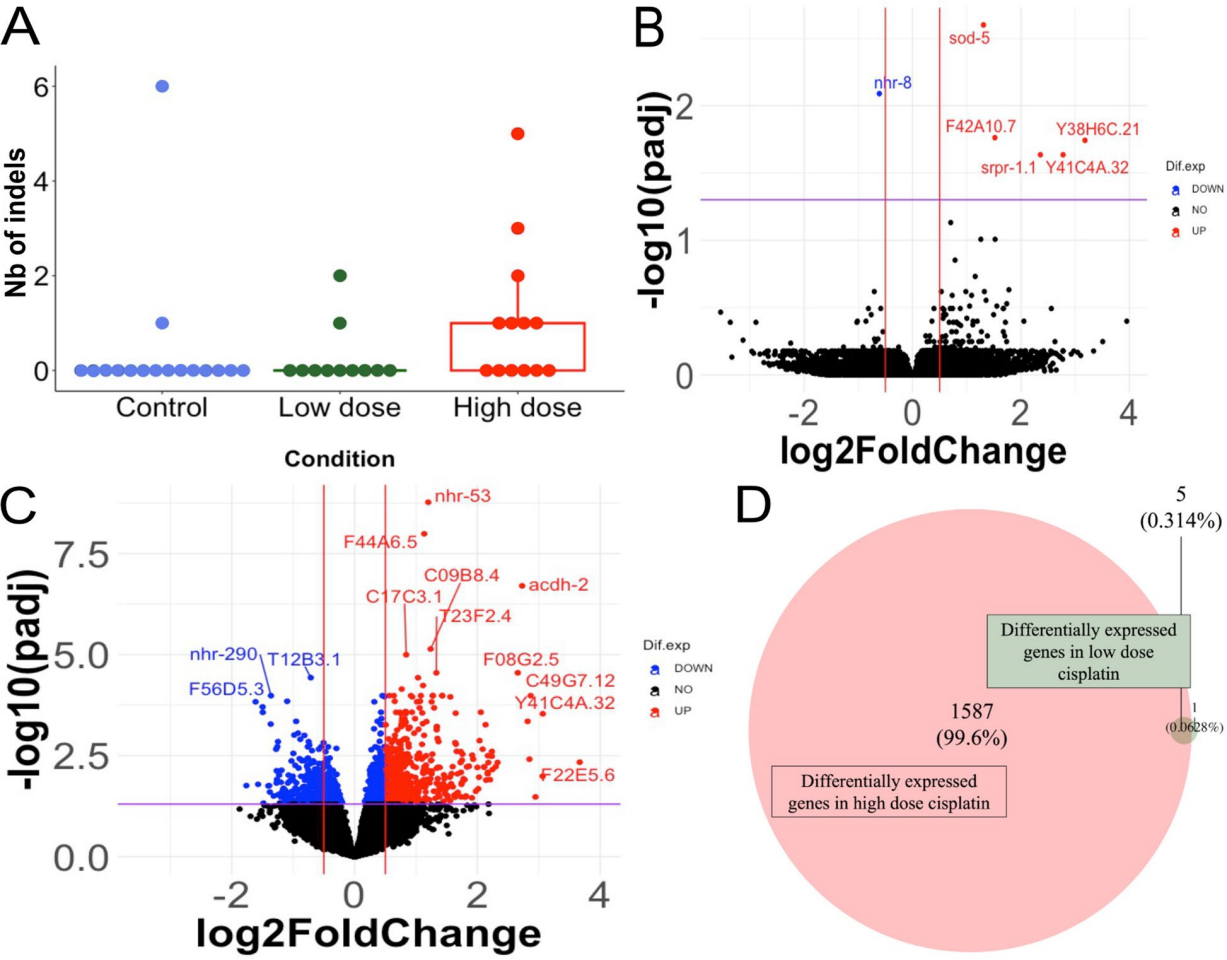
Effect of cisplatin on gene expression and DNA mutations. **A** Boxplot of the number of indels arising in each generation for the control condition (blue), cisplatin low dose (green) and cisplatin high dose (red). Each dot represents ATACseq data from a generation in the epimutation accumulation line with control: *N* = 16, low dose: *N* = 11, high dose: *N* = 13. Two lineages per condition were used. A significant increase in indels number was observed in high-dose cisplatin (Kruskal–Wallis rank-sum test (*p*-value = 0.05) followed by the Conover-Iman test: control vs. high dose, *p*-value = 0.01; control vs. low dose, *p*-value = 0.40; low dose vs. high dose, *p*-value = 0.03). **B** Volcano plot representing the gene expression changes in low-dose cisplatin compared to control. RNAseq data of each generation of the two lineages in each condition were used as replicates in DESeq2 giving control: *N* = 21, low dose: *N* = 20. *x*-axis = log2FoldChange of the genes; *y*-axis =  − log10(adjusted *p*-value). Both fold change values and adjusted *p*-values were calculated using DESeq2. Genes in red are significantly upregulated, genes in blue are significantly downregulated and genes in black showed no change in expression. **C** Volcano plot representing the gene expression changes in high-dose cisplatin compared to control. RNAseq data of each generation of the two lineages in each condition were used as replicates in DESeq2 giving control: *N* = 21, high dose: *N* = 19. *x*-axis = log2FoldChange of the genes; *y*-axis =  − log10(adjusted *p*-value). Both fold change values and adjusted *p*-values were calculated using DESeq2. Genes in red are significantly upregulated, genes in blue are significantly downregulated and genes in black showed no change in expression. **D** Venn diagram of differentially expressed genes in high-dose cisplatin compared to control (red), differentially expressed genes in low-dose cisplatin compared to control and genes differentially expressed in both conditions (low and high dose). Six genes were differentially expressed in the low-dose condition, 1592 in the high-dose condition and 5 genes shared differentially expression in the two conditions. Supporting information is available in the Excel file: “Additional file 9”

To investigate how epimutations might be affected by genotoxic stress, we established parallel lines of *C. elegans*, propagated under minimal selection, in control conditions, and exposed to either 75 µM or 150 µM cisplatin (henceforth low and high dose), selected such that the treatment had a notable effect on embryonic lethality but did not block propagation (Additional file 6: Fig. S2A). High-dose cisplatin led to a significant increase in the rate of indels (Fig. 2A and Additional file 7: Fig. S3), confirming that the treatment induced genotoxic stress, as expected based on the known mechanism of cisplatin in inducing DNA damage through monoadducts, inter- and, most prominently, intrastrand cross-links [39, 40]. Nevertheless, the total number of mutations induced over the course of the experiment was still relatively small (at around 13.5 SNPs and 7 indels by 20 generations); thus, the rate of mutation even under cisplatin exposure is still orders of magnitude lower than the expected rate of epimutations [9, 11]. No significant effect of the cisplatin exposures was observed on the rate of SNPs (data not shown).

Chronic exposure to cisplatin could induce gene expression changes directly, independent of epimutations. To identify these effects, we looked for genes that showed consistent changes in expression across all generations of the experiment. We identified 6 differentially expressed (DE) genes in low dose compared to control (Fig. 2B) and 1592 DE genes in high dose compared to control (DESeq2, Wald test, *p*-value < 0.05) (Fig. 2C). Five DE genes were shared between low- and high-dose conditions (Fig. 2D). Since only a few DE genes were observed in low dose, no relevant enriched function was identified during the GO term enrichment analysis. However, 279 significantly enriched functions in high-dose cisplatin condition compared to control were observed (Additional file 8: Table S5). The gene expression differences observed support the conclusion that cisplatin exposure induces genotoxic stress (Additional file 6: Fig. S2B).

We next investigated the potential of DNA mutations to be responsible for gene expression changes in our dataset. We examined the changes in gene expression that occurred relative to the first generation of each lineage, where changes in expression were unlikely to be caused by exposure to cisplatin. We identified genes showing both expression change and DNA mutation at the same generation (Table 1). No genes underwent both kinds of changes in the control condition. In the cisplatin low-dose condition, three genes had both expression changes and DNA mutation and only one gene in the cisplatin high-dose condition. Thus, only a small number of genes with expression changes over the course of the experiment were associated with DNA mutations (control: 0/11,497; low dose: 3/10,058; high dose: 1/9737). In addition, the two genes in the L1 lineage with both change in expression and DNA mutation had a change in expression at three different generations and a DNA mutation observed in only one of them, making a correlation between the DNA mutation and the change in expression rather unlikely.

**Table 1:** Genes showing both a change in the expression level and a DNA mutation

**Condition**	**Lineage**	**Gene (WB)**	**Gene**	**Gen. exp. chg**	** + /** −	**Gen. mut**	**Mut. type**
Low dose	L1	WBGene00019132	bath-19	2_10_16	+	16	Indel
Low dose	L1	WBGene00018024	F35A5.1	2_10_16	+	16	SNP
Low dose	L2	WBGene00012792	Y43D4A.6	20	+	20?	SNP
High dose	H2	WBGene00001839	hdl-1	18	−	18	SNP

Data were missing for DNA mutation identification at generations 18 and 20 for low-dose L2, but in the gene Y43D4A.6, the SNP appeared at generation 10 and up to generation 16; we thus assumed that it would also be observed in the subsequent generations if it is a true mutation. Supporting information for this table can be found in the Excel file: “Additional file 10”

The “ + / − ” column indicates if the observed change was overexpression ( +) or underexpression ( −)

*WB* WormBase identification number, *Gen. exp chg.* generations with gene expression change; *Gen. mut.* generations showing DNA mutation; *Mut. type*. type of mutation

In conclusion, the cisplatin treatment did impact the rate of DNA mutations with a correlation between an increase in dose and an increase in the number of new mutations observed. This result is not surprising as cisplatin is a genotoxic compound, and exposure to cisplatin had led to DNA mutational signatures in several organisms [41–43]. Despite the increase in the rate of DNA mutations, a correlation between DNA mutations and gene expression changes concerned only a very limited number of genes.

### Cisplatin exposure has a limited effect on the rate and spectrum of heritable gene expression changes

Having established that cisplatin exposure induced genotoxic stress, we sought to decipher whether the properties of epimutations were affected. Importantly, we wanted to specifically identify epimutations rather than direct transcriptional responses to cisplatin. We therefore investigated changes in gene expression that occurred in each individual generations, within the individual lineages and within the time course of the experiment.

**Fig. 3:**
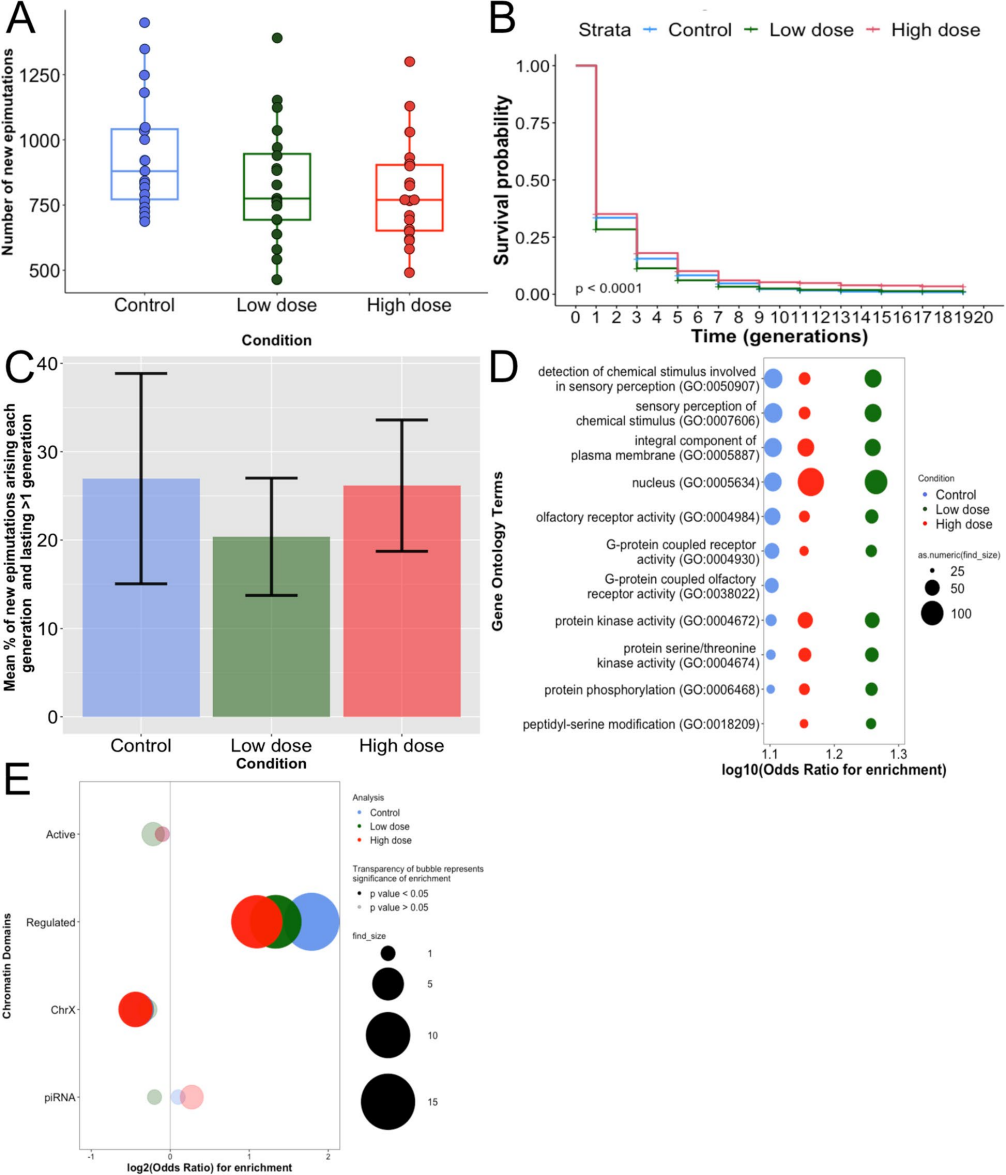
Effects of cisplatin exposure on gene expression epimutations. **A** Boxplot of the number of new RNA epimutations arising at each generation of the MA lines compared to the pre-mutation generation F0 and for each condition: control (blue), cisplatin low dose (green) and cisplatin high dose (red). Each dot represents a generation in a lineage, and two lineages were used for each condition with control: *N* = 19, low dose: *N* = 20 and high dose: *N* = 19. No significant difference was observed between the conditions (Kruskal–Wallis rank-sum test, *p*-value = 0.11). **B** Survival curves representing the new RNA epimutations duration in each exposure condition: control (blue), cisplatin low dose (green) and cisplatin high dose (red). Curves were computed using the two lineages per condition as biological replicates. We observed a significant difference between the three conditions with an increased duration of epimutations in high dose and a decreased duration in low dose compared to control (log-rank test, *p*-value < 1e − 04). **C** Barplot of the mean percentage of new RNA epimutations that lasted more than one generation compared to the total epimutations arising each generation. Data are presented by condition: control (blue), cisplatin low dose (green) and cisplatin high dose (red). The means were calculated per generation from generation 2 to 18 with *N* = 9 for each condition. The mean percentages of lasting epimutations compared to all epimutations were 26.9% for control, 20.4% for low dose and 26.2% for high dose. No significant difference was observed between conditions. **D** Bubble plot illustrating ontology term enrichment of RNA epimutations lasting more than one generation in control (blue), cisplatin low dose (green) and cisplatin high dose (red) compared to gene without epimutation. Enrichments were calculated using data from generation 2 to 18 used as technical replicates, and two lineages per condition were used as biological replicates. Enrichment was calculated with *χ*.^2^ test. The top 10 results per condition are shown. The *x*-axis shows the log10(*χ*) for enrichment. The *y*-axis shows the ontology terms. All displayed ontology terms were significantly enriched. **E** Bubble plot showing the distribution of lasting RNA epimutations in control (blue), cisplatin low dose (green) and cisplatin high dose (red). Data from generation 2 to 18 were used as technical replicates, and two lineages per condition were used as biological replicates. The *y*-axis displays the constitutive chromatin domains investigated. Active chromatin domains correspond to domains enriched in H3K36me3 mark and regulated domains to domains enriched in H3K27me3[44]. The *x*-axis shows the log2(odds) of enrichment. Odds ratio and *p*-values were calculated using Fisher’s exact test with Bonferroni correction. *p*-value cut-off for significance is 0.05. Supporting data are provided in the Excel file: “Additional file 14”

We first observed that the number of new epimutations affecting gene expression was not significantly different between control and cisplatin-treated lines (Kruskal–Wallis rank sum test, *p*-value = 0.112) (Fig. 3A and Additional file 11: Fig. S4A). The duration of epimutations affecting gene expression showed a significant difference across conditions (log-rank test, *p*-value < 1e − 4). Epimutations tended to last slightly longer under conditions of high-dose cisplatin and slightly shorter in low-dose cisplatin relative to control conditions (Fig. 3B and Additional file 11: Fig. S4B). However, these differences were small, as demonstrated by an analysis of the fraction of changes in the expression seen in each generation that were inherited, which was similar between all conditions (Fig. 3C and Additional file 11: Fig. S4C).

We next investigated whether the spectrum of genes affected by epimutations was modified by cisplatin exposure. Using Gene Ontology analysis, we identified some significantly enriched GO terms that were specific to cisplatin exposure (Additional file 12: Table S6); however, there was a strong overlap between the top 10 enriched GO terms in all conditions (Fig. 3D and Additional file 11: Fig. S4D), indicating that cisplatin exposure did not result in large shifts in genes affected by epimutations. Cisplatin-inducing DNA damage responsible for cell death, we specifically looked at the epimutation rate in the genes involved in apoptosis. The results showed no significant difference between conditions in the occurrence of new epimutations targeting apoptotic genes (Additional file 13: Fig. S5).

We also looked at the chromatin environment of genes affected by epimutations (Fig. 3E and Additional file 11: Fig. S4E). Under control conditions, epimutations were enriched in regulated regions rich in H3K27me3 and depleted from active regions enriched in H3K36me3 and from the X chromosome which was consistent with our previous results [11]. Cisplatin exposure did not alter these trends, supporting the conclusion that cisplatin exposure did not change the spectrum of epimutations.

### Cisplatin exposure does not affect the rate or spectrum of sncRNA-mediated epimutations

**Fig. 4:**
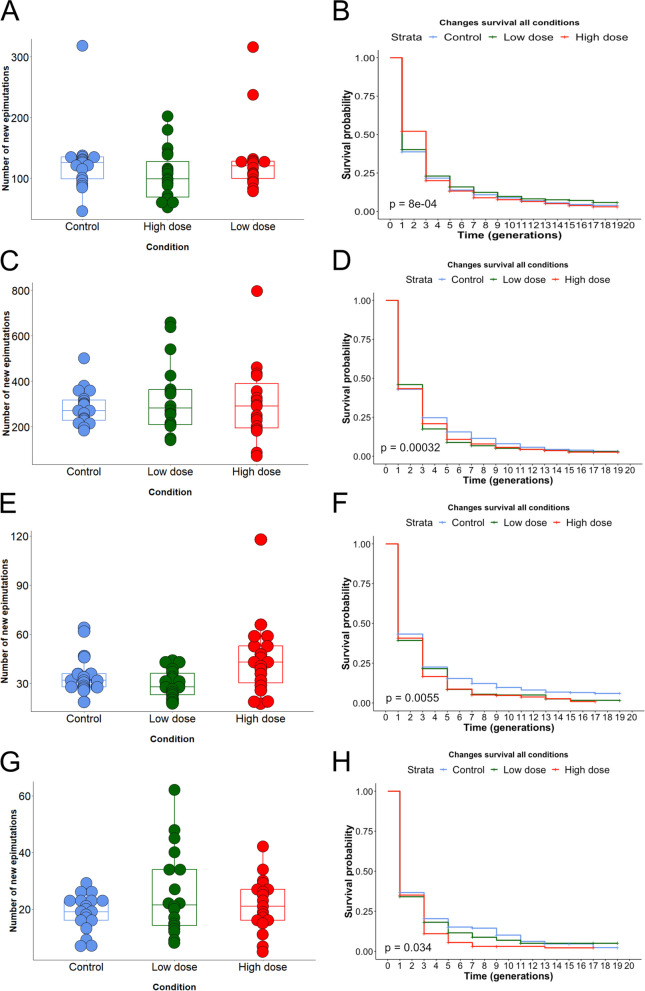
Effects of cisplatin exposure on sncRNA epimutations. **A** Boxplot of 22G-RNA epimutations rate for each generation of the MA lines compared to the pre-mutation generation F0 and for each condition: control (blue), cisplatin low dose (green) and cisplatin high dose (red). No significant difference was observed between conditions (Kruskal–Wallis rank-sum test, *p*-value = 0.29). **B** Survival curves representing the new 22G-RNA epimutations duration in each exposure condition: control (blue), cisplatin low dose (green) and cisplatin high dose (red). We observed a significant difference between the three conditions (log-rank test, *p*-value = 8e − 04), but the difference was not dose-dependent. **C** Boxplot of piRNA epimutation rate for each generation of the MA lines compared to the pre-mutation generation F0 and for each condition: control (blue), cisplatin low dose (green) and cisplatin high dose (red). No significant difference was observed between the conditions (Kruskal–Wallis rank-sum test, *p*-value = 0.90). **D** Survival curves representing the new piRNA epimutations duration in each exposure condition: control (blue), cisplatin low dose (green) and cisplatin high dose (red). We observed a significant difference between the three conditions (log-rank test, *p*-value = 3.2e − 04) with slightly higher survival changes in epimutation in the control condition. **E** Boxplot of miRNA epimutations rate for each generation of the MA lines compared to the pre-mutation generation F0 and for each condition: control (blue), cisplatin low dose (green) and cisplatin high dose (red). No significant difference was observed between the conditions (Dunn’s Kruskal–Wallis multiple comparison, control vs. LD: *p*-value = 0.28; control vs. HD: *p*-value = 0.25). **F** Survival curves representing the new miRNA epimutations duration in each exposure condition: control (blue), cisplatin low dose (green) and cisplatin high dose (red). We observed a significant difference between the three conditions (log-rank test, *p*-value = 5.5e − 03) with slightly higher survival changes in epimutation in the control condition. **G** Boxplot of 26G-RNA epimutations rate for each generation of the MA lines compared to the pre-mutation generation F0 and for each condition: control (blue), cisplatin low dose (green) and cisplatin high dose (red). No significant difference was observed between the conditions (Kruskal–Wallis rank-sum test, *p*-value = 0.52). **H** Survival curves representing the new 26G-RNA epimutations duration in each exposure condition: control (blue), cisplatin low dose (green) and cisplatin high dose (red). We observed a significant difference between the three conditions (log-rank test, *p*-value = 0.03 with slightly higher survival changes in epimutation in the cisplatin low-dose condition*.* For **A**, **C**, **E** and **G**, each dot represents a generation, and data from two lineages per condition were combined giving a number of replicates of *N* = 19 for control, *N* = 18 for low dose and *N* = 19 for high dose. For **B**, **D**, **F** and **H**, each curve was built using the two lineages per condition as biological replicates (*N* = 2 for each condition). Supporting data for the whole figure can be found in the Excel file: “Additional file 18”

Small non-coding RNAs are well established as carriers of transgenerational epigenetic inheritance in *C. elegans* [17]. We first investigated 22G-RNAs, as they are the major source of epimutations, and found no difference between control and cisplatin-treated lines in terms of epimutation rate (Fig. 4A and Additional file 15: Fig. S6A) nor a cisplatin dose-dependent change of epimutations duration (Fig. 4B and Additional file 15: Fig. S6B).

We previously found that 22G-RNA epimutations were overlapping with changes in gene expression and genes showing heritable changes in gene expression were significantly enriched for simultaneous 22G-RNA-based epimutations, especially for genes linked to xenobiotic defence [11]. Therefore, we investigated the association between 22G-RNAs and gene expression changes in our control and cisplatin-treated lines. Amongst genes that had inherited RNA expression changes and were targeted by 22G-RNA epimutations, we calculated the percentage of which that had simultaneous inherited 22G-RNA epimutations, the percentage of which that had non-inherited 22G-RNA epimutations and the percentage of that had non-simultaneous 22G-RNA epimutations, and thus for each condition (Additional file 16: Fig. S7A, left stacked-barplots in each panel). We did the same among the genes that had non-inherited RNA expression changes and were targeted by 22G-RNA epimutations (Additional file 16: Fig. S7A, right stacked barplots in each panel). We found quite similar results for both control and low-dose cisplatin conditions (37.5% of genes with inherited expression change and targeted 22G-RNA epimutation had simultaneous and inherited 22G-RNA epimutations in control, 33.3% in low dose) with a decrease in the percentage of genes (20%) with both simultaneous and inherited expression changes and 22G-RNA epimutations for cisplatin high dose condition.

Previously, we demonstrated that gene expression changes that were inherited transgenerationally were more likely to be associated with epimutations in 22G-RNAs. We recapitulated this observation in control and low-dose and high-dose cisplatin using an association analysis. However, the enrichments were reduced in high-dose cisplatin, suggesting that the association between 22G-RNA changes and gene expression changes was weakened by the exposure to cisplatin (Additional file 17: Table S7). This may indicate a generalised uncoupling of gene expression and 22G-RNA level under conditions of stress perhaps due to increased noise in gene expression in 22G-RNA biogenesis.

Our next step was to look at piRNAs, involved in transgenerational epigenetic inheritance in *C. elegans* [45], and miRNAs that have been suspected to play a role in epigenetic inheritance in medaka fish [46] or in rats [47] after exposure to chemicals. In addition, we had found in a previous study that both miRNAs and piRNAs could be inherited transgenerationally [11]. The results revealed no significant difference due to the cisplatin treatments for both piRNA epimutation rate (Fig. 4C and Additional file 15: Fig. S6C) and epimutation duration (Fig. 4D and Additional file 15: Fig. S6D). Similarly, we observed no difference in the total number of new miRNA epimutations per generation between control and treated lines (Fig. 4E and Additional file 15: Fig. S6E) or in the duration of epimutations (Fig. 4F and Additional file 15: Fig. S6F).

Finally, we investigated 26G-RNAs that are small interfering RNAs (siRNAs) enriched in the germline and involved in the 22G-RNAs synthesis [48]. Once again, no overall significant difference in epimutations rate (Fig. 4G and Additional file 15: Fig. S6G) or the epimutations duration (Fig. 4H and Additional file 15: Fig. S6H) between conditions was identified.

### Cisplatin exposure affects the rate of tRNA epimutations

**Fig. 5:**
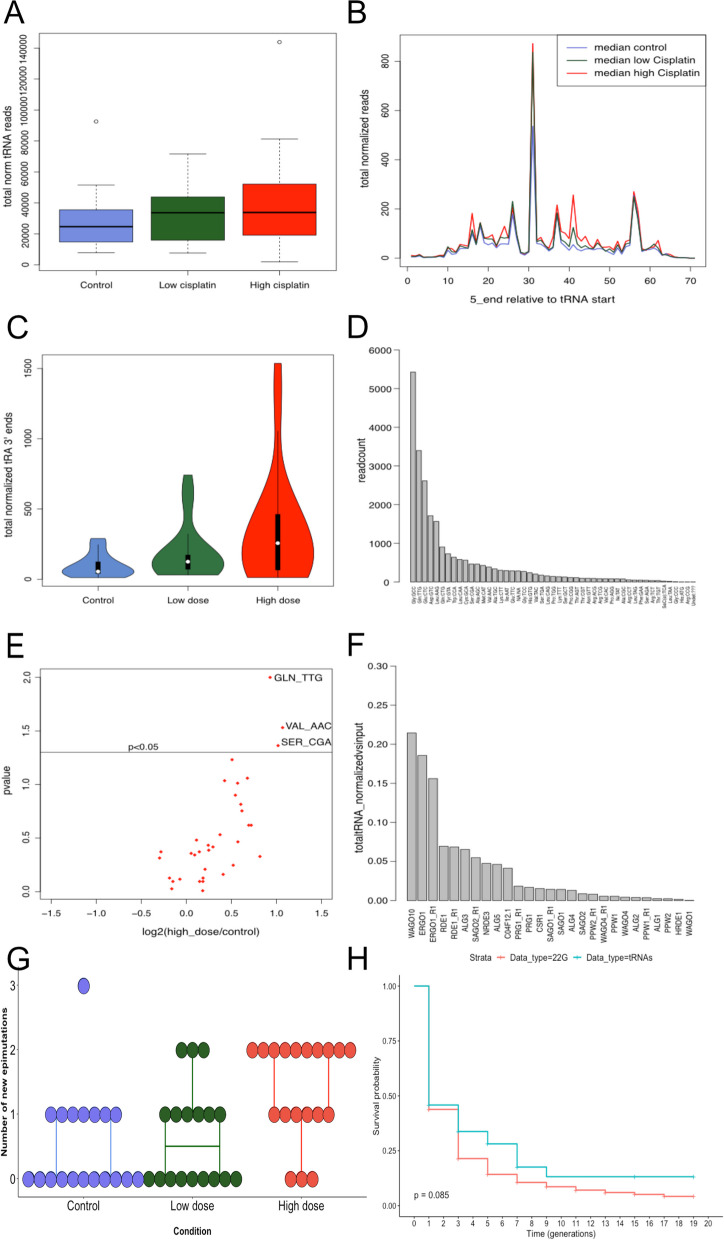
Cisplatin effects on tRNAs. **A** Total normalised reads that mapped to tRNAs in each condition: control condition (blue), cisplatin low-dose condition (green) and cisplatin high-dose condition (red). Data from generations were used as technical replicates and the two lineages per condition as biological replicates, i.e. for control: *N* = 21, for low dose: *N* = 20 and for high dose: *N* = 21. **B** The total normalised reads represented according to their mapping positions on the respective tRNA sequence and in each different condition: control condition (blue), cisplatin low-dose condition (green) and cisplatin high-dose condition (red). Data from generations were used as technical replicates and the two lineages per condition as biological replicates, i.e. for control: *N* = 21, for low dose: *N* = 20 and for high dose: *N* = 21. **C** Violin plot of the total normalised tRNAs mapping specifically to the 3′ half of the tRNAs in the different conditions: control condition (blue), cisplatin low-dose condition (green) and cisplatin high-dose condition (red). Data from generations were used as technical replicates and the two lineages per condition as biological replicates, i.e. for control: *N* = 21, for low dose: *N* = 20 and for high dose: *N* = 21. A significant increase was observed in both low- and high-dose cisplatin, with a trend towards increased reads in high-dose cisplatin (*p* < 2e − 16, Jonckheere test for ordered medians). **D** Bar plot showing the number of read counts associated to each kind of tRNAs. Data from all generations and all lineages were used giving 62 replicates. The tRNAs with the most associated fragments were GlycineGCC, GlutamineTTG and Glutamic acidCTC. **E** Volcano plot of the expression change between high dose and control (in log2(fold change)) for the different kinds of tRNAs. Data from generations were used as technical replicates and the two lineages per condition as biological replicates, i.e. for control: *N* = 21 and for high dose: *N* = 21. Points above the *p* < 0.05 line show a significant expression change for these corresponding tRNAs. Adjusted *p*-values were calculated using DESeq2. **F** Bar plot of the total normalised 3′ halves tRNAs reads and their association to each AGO protein. Each replicate per AGO was used. Both Ergo1 and Wago10 showed a high enrichment in tRNA 3′ halves relative to control Ips. **G** Boxplot of the number of new epimutations affecting tRNA fragments arising at each generation of the MA lines compared to the pre-mutation generation F0 and for each condition: control (blue), cisplatin low dose (green) and cisplatin high dose (red). Data from generations were used as technical replicates and the two lineages per condition as biological replicates, i.e. for control: *N* = 19, for low dose: *N* = 18, and for high dose: *N* = 19. A significant increase was observed in cisplatin high-dose condition (Kruskal–Wallis rank sum test followed by pairwise Wilcoxon test with Bonferroni correction: HD vs. C: *p*-value = 3.6e − 03, HD vs. LD: *p*-value = 0.02). **H** Survival curves representing the duration of epimutations in 22G-RNAs (red, *N* = 6) and 3′ halves tRNA fragments (blue, *N* = 6). No significant difference was observed between the two types of snRNAs (log-rank test, *p*-value = 0.09). Supporting data for the whole figure are available in the Excel file: “Additional file 23”

In the course of our analyses of sncRNAs, we noticed that there appeared to be more reads mapping to tRNA loci under cisplatin conditions (Fig. 5A and Additional file 19: Fig. S8A). We were intrigued by this because tRNA halves are known to be abundant in mammalian cells and have been implicated in intergenerational epigenetic transmission through mammalian sperm [4, 47, 49–51]. We therefore decided to investigate whether tRNA might be induced by cisplatin treatment. We mapped sncRNAs to tRNAs, demonstrating that the predominant initiation point for sncRNAs coincided with the 3′ half of the tRNA (Fig. 5B and Additional file 19: Fig. S8B). Taken across all generations, there was a significant increase in the reads mapping specifically to the 3′ half of the tRNA in both low- and high-dose cisplatin, and a trend towards increased reads in high-dose cisplatin (*p* < 2e − 16, Jonckhere test for ordered medians, Fig. 5C and Additional file 19: Fig. S8C). The most abundant tRNA fragments in all conditions combined were GlycineGCC, GlutamineTTG and Glutamic acidCTC (Fig. 5D). Three tRNAs showed a significant increase in expression in cisplatin high dose: GlutamineTTG, ValineAAC and SerineCGA (DESeq2: adjusted *p*-value < 0.05, Fig. 5E).

According to the canonical viewpoint, in order for small ncRNAs to function in gene expression control they must associate with proteins of the Argonaute family (AGOs) [52]. We therefore wondered whether tRNA fragments might similarly be associated with AGOs in *C. elegans* and thus function similarly to other types of small non-coding RNAs such as 22G-RNAs. To investigate this possibility, we took advantage of the previously published dataset which performed immunoprecipitation followed by RNA sequencing for all *C. elegans* AGOs [18] to test whether tRNA 3′ halves could be found associated with specific AGOs. Most AGO immunoprecipitations were strongly depleted for tRNA fragments. Both ERGO1 and WAGO10 showed a ~ 20% enrichment in tRNA 3′ halves relative to control IPs (Fig. 5F). Importantly, these enrichments, whilst clearly greater than any of the other AGOs, are still weak relative to the previously described substrates [18]. We next tested whether ERGO-1 or WAGO-10 might contribute to stabilising tRNA fragments. We investigated the levels of tRNA fragments in mutants lacking e*rgo-1* or *wago-10*. For comparison, we also examined rde-1 mutants, which had the third highest enrichment in IPs (Fig. 5F) but had negligible overall enrichment. Neither *ergo-1* nor *rde-1* showed a decrease in tRNA fragment levels. *wago-10* mutants showed a modest decrease in tRNA fragment levels, corresponding to a significant decrease in Glutamate tRNA fragments (Wilcoxon test, *p* < 0.05, Additional file 20: Fig. S9A and B). Together, these data suggested that ERGO-1 and WAGO-10 may bind some tRNA fragments, and WAGO-10 additionally may stabilise these fragments. However, the modest nature of the enrichment indicated that this is unlikely to be the primary mode of action of tRNA 3′ halves.

We next tested whether tRNA 3′ halves induced by cisplatin treatment showed the characteristics of epimutations, in other words showing heritability over a short number of generations similar to 22G-RNAs. We first observed a significant increase in the rate of tRNAs new epimutations in cisplatin high dose (Kruskal–Wallis rank sum test followed by the pairwise Wilcoxon test with Bonferroni correction: HD vs. C: *p*-value = 4e − 3; HD vs. LD: *p*-value = 0.02) (Fig. 5G and Additional file 19: Fig. S8D), suggesting that the overall increase in reads from tRNA halves is due to increased fluctuations in their expression rather than a consistent change. We later compared the survival of 22G-RNAs and tRNAs, which demonstrated that tRNAs and 22G-RNAs were inherited for a similar duration (Fig. 5H). We then created a table with the number and maximal duration of epimutations by tRNA type (Additional file 21: Table S8). Several tRNAs were epimutated only in the presence of cisplatin (TrpCCA, SerCGA, LeuAAG, GlyGCC, GlnCTG, AspGTC and AlaAGC). The two tRNAs with the highest number of epimutations were both found in high-dose cisplatin and were GlyGCC and GlnTTG (Additional file 22: Fig. S10A and B).

However, the epimutations that occurred did not last longer than those occurring in control conditions (log-rank test, *p*-value = 0.081) (Additional file 22: Fig. S10C). Thus, cisplatin exposure led to the increased fluctuations in the expression of tRNA halves which were not transmitted between generations.

### Fluctuations in tRNA 3′ halves are associated with changes in expression of potential target genes

**Fig. 6:**
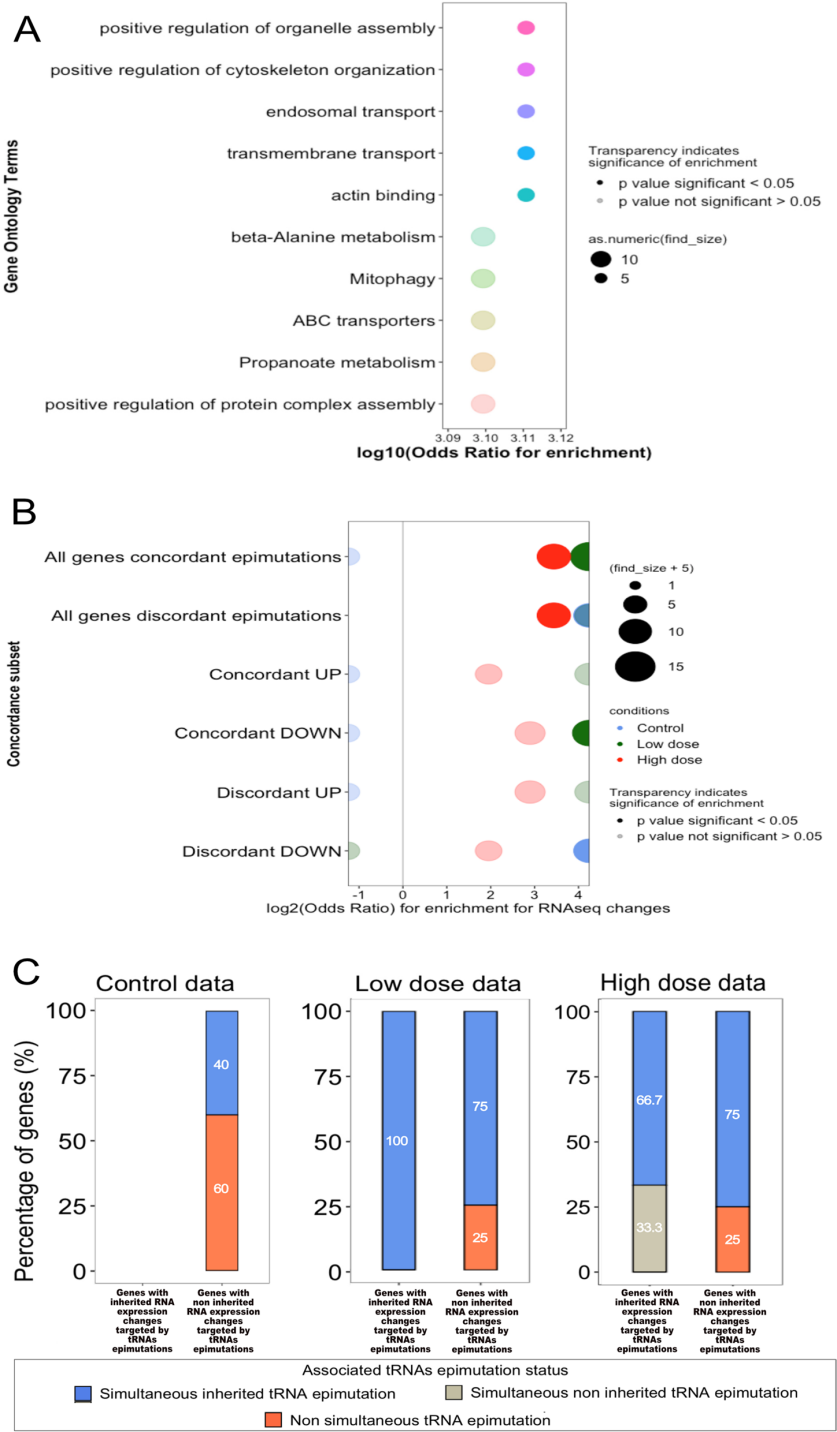
Correlation gene expression changes and tRNA epimutations. **A** Bubble plot showing the ontology term enrichment of genes associated with tRNAs. Enrichment calculated with *χ*.^2^ test; top 10 results shown. The *x*-axis shows the log10(*χ*) for enrichment. The *y*-axis shows the ontology terms. Data from all lineages and all conditions were combined, i.e. *N* = 6. **B** Bubble plot of concordance (simultaneous and direction matched) or discordance (simultaneous but direction unmatched) of gene expression changes and tRNA epimutations in control condition (blue dots), cisplatin low dose (green dots) or cisplatin high dose (red dots). Two lineages per condition were used as biological replicates. The *y*-axis shows the association tests. The *x*-axis shows the log2(odds ratio) of gene expression changes to be associated with tRNA epimutation within each association. Odds ratios and *p*-values were calculated using Fisher’s exact test. **C** Stacked barplots representing the proportion of genes with inherited (left bar) of non-inherited (right bar) RNA expression changes targeted by tRNA epimutations in the control condition (left panel), cisplatin low-dose condition (middle panel) and cisplatin high-dose condition (right panel). Two lineages per condition were used as biological replicates. In blue, the percentage of genes with simultaneous inherited tRNA epimutations; in grey, the percentage of genes with simultaneous non-inherited tRNA epimutations; and in orange, the percentage of genes with non-simultaneous tRNA epimutations. Supporting data for the whole figure is available in the Excel file: “Additional file 25”

Having established that tRNA 3′ halves show increased fluctuations in their levels under conditions of cisplatin exposure, we wondered whether this might have any consequences for gene expression. We searched for potential targets of tRNAs. Whilst there were no genes with any regions demonstrating perfect complementarity to tRNA 3′ halves, we identified 38 genes with up to 2 mismatches. Amongst several broad categories that were enriched, we noted that these genes were enriched for ABC transporters, a large family of ATP-driven trans-membrane transport enzymes, which we had previously identified as being enriched for epimutations in gene expression [11] (Fig. 6A). We then more specifically looked at genes involved in apoptosis. Only one of them was a potential tRNA target: vps-16, but no associated tRNA 3′ halve epimutations were observed in any of the exposure conditions (Additional file 24: Table S9).

In all conditions, changes in the expression of these genes were strongly associated with simultaneous changes in the expression of tRNAs (Table 2, test 1). The association was reduced in high-dose cisplatin. However, due to the higher number of tRNA fragments showing changes, the total number of genes showing changes in both tRNA fragments and gene expression was 1.4-fold higher in high-dose cisplatin than in the control condition. In the control condition, the association between gene expression change and tRNA 3′ halves epimutation was mostly discordant such that increased tRNA levels were associated with reduced gene expression (Fig. 6B). However, tRNA fragment increases were equally likely to be associated with up and downregulation of the genes under both high- and low-dose cisplatin exposure.

**Table 2:** Stepwise analysis of the association of gene expression changes and tRNA epimutations in the different exposure conditions

Condition	Test	Association tested	Background	Odds ratio	*p*-value
Control	1	Gene exp. change and tRNA epimutations	All genes	Infinite	9.58e − 04
Control	2	Inherited gene exp. change and tRNA epimutations	All genes with RNA-seq changes	0.34	0.03
Control	3	Inherited gene exp. change and simultaneous tRNA epimutations	All genes with RNA-seq changes	0	1
Control	4	Inherited gene exp. change and simultaneous tRNA epimutations	All genes with RNA-seq changes and tRNA epimutations	0	1
Low dose	1	Gene exp. change and tRNA epimutations	All genes	Infinite	8.86e − 05
Low dose	2	Inherited gene exp. change and tRNA epimutations	All genes with RNA-seq changes	0.65	1.00e + 00
Low dose	3	Inherited gene exp. change and simultaneous tRNA epimutations	All genes with RNA-seq changes	0.23	7.30e − 03
Low dose	4	Inherited gene exp. change and simultaneous tRNA epimutations	All genes with RNA-seq changes and tRNA epimutations	Infinite	1.00e + 00
High dose	1	Gene exp. change and tRNA epimutations	All genes	19.78	2.81e − 04
High dose	2	Inherited gene exp. change and tRNA epimutations	All genes with RNA-seq changes	1	1
High dose	3	Inherited gene exp. change and simultaneous tRNA epimutations	All genes with RNA-seq changes	1.61	0.70
High dose	4	Inherited gene exp. change and simultaneous tRNA epimutations	All genes with RNA-seq changes and tRNA epimutations	Infinite	1

Cells with *p*-value < 0.05 indicate significantly increased odds of association, and cells with *p*-value > 0.05 in grey indicate no significant association. Odds ratios and *p*-values were calculated with Fisher’s exact test. Data from the two lineages for each condition were combined (*N* = 2)

We then looked at the percentage of genes with inherited expression change that were also targeted by changes in tRNA 3′ halves levels in each condition (Fig. 6C). As for all genes with both gene expression and tRNA change, the percentage of genes with inherited gene expression change and simultaneous tRNA changes was lower in high dose than in the two other conditions (Table 2, tests 2–4).

**Fig. 7:**
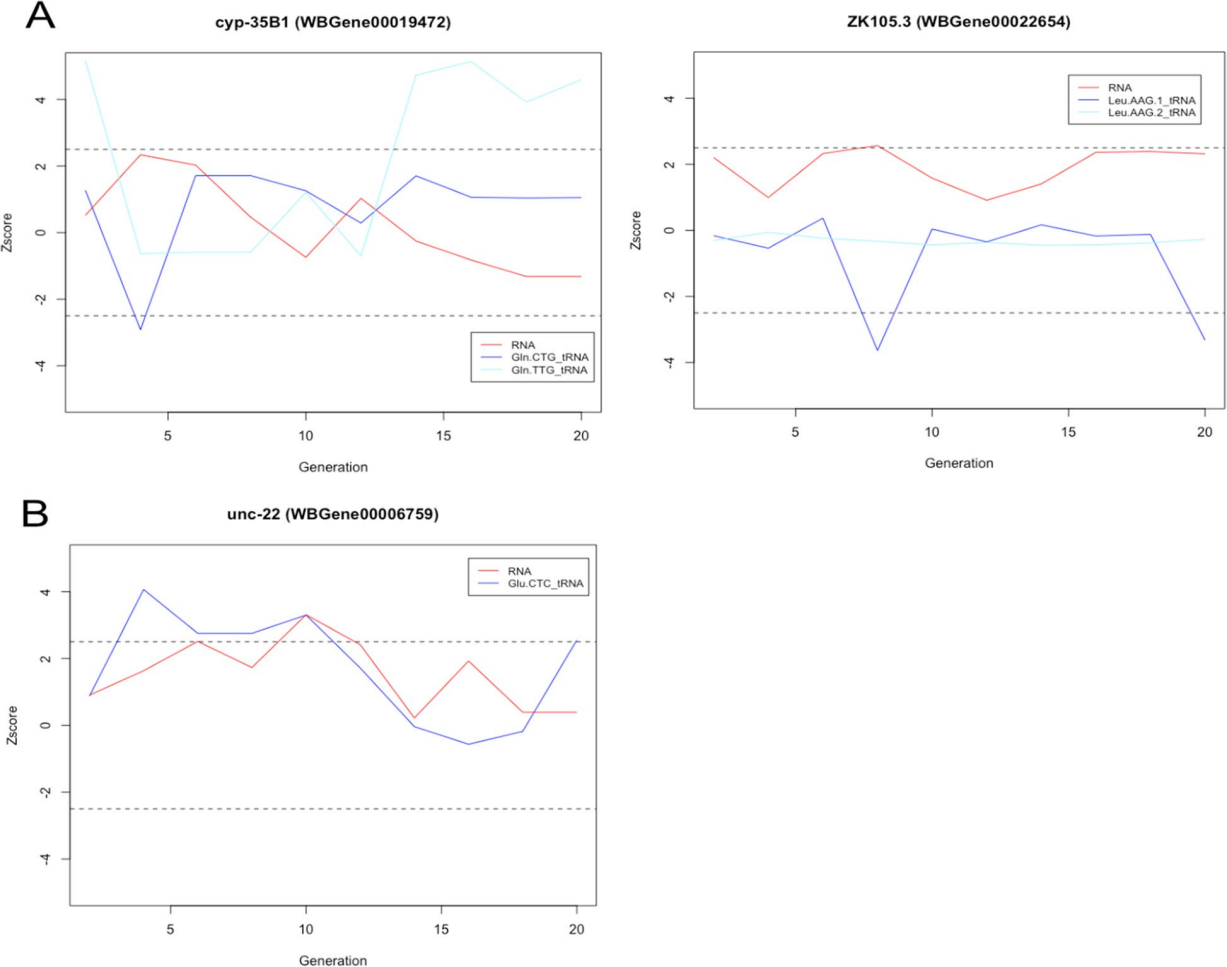
**A** Two examples of genes showing an anticorrelation between gene expression change and tRNA 3′ halves epimutation. The *x*-axis represents the generation. The *y*-axis shows the *z*-score. The red line represents the gene expression, and the blue line represents the tRNA 3′ halves. **B** One example of a gene showing a correlation between gene expression change and tRNA 3′ halves epimutation. The *x*-axis represents the generation. The *y*-axis shows the *z*-score. The red line represents the gene expression, and the blue line represents the tRNA 3′ halves. Each example comes for a single lineage (*N* = 1). Supporting data for the whole figure can be found in the Excel file: “Additional file 26”

Interestingly, on average, 45.8% of changes in tRNA level were associated with a simultaneous gene expression change, compared to 8% for 22G-RNAs (Table 3). Moreover, some of the tRNA changes were associated with changes in the gene expression that persisted for multiple generations (Fig. 7).

**Table 3:** Comparison of odds of association between 22G-RNA or tRNA epimutations and gene expression changes

Condition	Association tested	*X* = 22G-RNAs	*X* = tRNAs	Odds ratio	*p*-value
Control	Genes with exp. change + simultaneous X epimutation	75	2	0.66	0.64
Control	Genes with exp. change only	912	16		
Low dose	Genes with exp. change + simultaneous X epimutation	73	4	0.16	9.30e − 03
Low dose	Genes with exp. change only	936	8		
High dose	Genes with exp. change + simultaneous X epimutation	65	6	0.10	2.96e − 04
High dose	Genes with exp. change only	851	8		

Cells with *p*-value < 0.05 indicate the significantly increased odds of association for tRNAs compared to 22G-RNAs. Odds ratios and *p*-values were calculated with Fisher’s exact test. Data from the two lineages for each condition were combined (*N* = 2)

## Discussion

As previously discussed in the literature [41–43], our results confirmed that cisplatin exposure led to genotoxic stress with an increase in the rate of random mutations. We wanted to investigate if cisplatin might also affect the rate, spectrum, or duration of epimutations. Our results showed that epimutations were not strongly affected by cisplatin exposure, as identified changes were similar in both control and cisplatin conditions. However, cisplatin tends to increase the expression of reads mapping to 3′ tRNA halves. We discuss these results in terms of their significance for the contribution of epimutations to evolutionary processes and potential new insights into the biology of small non-coding RNAs in *C. elegans*.

Previous work has explored the rate, spectrum and stability of epimutations in plants [13–15, 53, 54] and *C. elegans* [9, 11] in the absence of selection, demonstrating that epimutations arise much more frequently than genetic changes involving DNA sequence alterations but have a limited duration. This suggests that epimutations are unlikely to contribute significantly to neutral evolution over long periods of time [14, 55]. However, this still leaves open the possibility that epimutations may contribute to evolution under conditions of strong selection [56]. The selection pressure would have to be strong enough to outweigh the limited half-life of any beneficial epimutation due to its limited stability. A key unknown in this discussion, nonetheless, is whether the rate of occurrence, the stability or the type of epimutations that occur is independent of the nature of the selective pressure itself. Our results pertain to this assumption, using genotoxic stress as an example of selective pressure. We measured the rate, stability and spectrum of epimutations in both gene expression and small non-coding RNAs, the pre-eminent molecular mechanism that gives rise to transgenerational epigenetic inheritance in *C. elegans* in the absence of selection, whilst applying genotoxic stress through cisplatin exposure. Crucially, under these conditions, we saw marginal changes in the global parameters of gene expression and small non-coding RNA epimutations. This is despite the fact that there was an increased rate of DNA sequence mutations. Indeed, the levels of DNA sequence mutation were extremely low in control conditions, so even the observed increase of 5 ~ fold in mutations would be unlikely to explain a substantial fraction of the epimutations, although trans-acting mutations can never be entirely ruled out as a source of epigenetic changes. This is an important result for evaluating the potential for such kind of epimutations to stimulate evolution as it shows that, at least for cisplatin exposure, epimutations are neither increased in overall rate nor directed to specific regions of the genome that might have adaptative consequences. The idea that epimutations might respond to environmental changes and thus provide particular significance in adaptation has been frequently proposed [57–60], and some authors have argued that this might operate in plants [61]. Our results argue against this proposal, at least in the case of small non-coding RNA-mediated epimutations in cisplatin exposure in *C. elegans*. Systematic analyses of epimutations in the absence of selection under different stresses would be required to comprehensively evaluate this idea.

Whilst we did not observe changes in the parameters of epimutations in response to cisplatin, we did observe changes in the levels of tRNA-derived small non-coding RNAs. Importantly, despite our observation that there was an increased rate of genetic changes under conditions of cisplatin exposure, these changes occurred independently in all four lines exposed to cisplatin which means that a mutation in a hypothetical trans-acting regulator of tRNAs is unlikely to be responsible for this. Work across a range of model systems has identified tRNA-derived small RNAs as an abundant component of the cellular small non-coding RNA fraction [62–67]. Most commonly, these are either 5′ or 3′ halves and arise through cleavage of the tRNA by several enzymes, most prominently RNAses [68–73]. Even if this appears to happen constitutively in mammalian cultured cells [74], tRNA fragments are further induced by cellular stress, including oxidative stress [75] and infection [76].

Despite their widespread conservation [77], tRNA fragments have not been extensively characterised in *C. elegans*. Our results demonstrate a clear increase in the levels of tRNA 3′ halves associated with exposure to cisplatin. The fact that RNA 3′ halves become much more abundant upon genotoxic stress may explain why tRNAs were not previously noted as abundant features of *C. elegans* small RNA populations. Interestingly, one of the few publications describing tRNA fragments in *C. elegans* demonstrated an increased abundance of 3′ tRNA fragments in aged worms [78], which supports the idea that they may be rare in unstressed worms but increase dramatically under certain stressful conditions. Moreover, this is consistent with their induction by cellular stress in mammalian cells.

What might be the function of the increased levels of tRNA halves in response to cisplatin? Stress-induced cleavage of tRNAs in mammalian cells is associated with translational repression [75, 79], and this may be a function of tRNA cleavage in cisplatin-exposed *C. elegans*. Indeed, the fact that the changes in tRNA fragment levels are highly variable across different cisplatin-exposed lines even within the same lineage suggests a response to cellular stress, potentially shutting down translation in cells that experience particularly high levels of damage. It may also be the case that the tRNA halves themselves have a direct function in gene expression control [80]. The association of tRNA fragments with Argonaute proteins has been documented in mammalian cells [77, 81–83] and plants [62, 84]; however, whether this results in gene expression changes is still debated. We determined a significant tendency for the levels of tRNAs 3′ halves to be associated with changes in the expression of potential targets; however, this often involved concordant changes, which argues against an obvious silencing role. Interestingly, further, the association between tRNA fragment levels and gene expression changes was reduced under conditions of cisplatin exposure, which may indicate that the majority of tRNA fragments do not affect gene expression and so increased fluctuations in tRNA levels weaken the association. Importantly, the small (~ 20%) enrichment in ERGO-1 and WAGO-10 immunoprecipitations and the small contribution that WAGO10 made to stabilising tRNA fragments suggest that Argonaute binding is unlikely to be the dominant mode whereby tRNA functions. tRNA fragments, however, may bind to other proteins to bring about gene expression changes. Our discovery that cisplatin induces tRNA fragments may enable *C. elegans* to be developed in the future as a model for investigations of the mechanistic basis of tRNA fragments in gene expression control.

Our discovery that tRNA fragments appear to be induced by cisplatin exposure in *C. elegans* is interesting in light of several recent studies showing that tRNA fragments can be transmitted intergenerationally through sperm in rodents [4, 51, 85–88]. Although alterations in tRNA fragment levels that we observed upon cisplatin treatment were mostly transient and not inherited transgenerationally, we did observe a small number of examples of epimutations involving tRNA fragments and their targets. Mechanistically, transgenerational epigenetic inheritance of small non-coding RNAs in *C. elegans* relies on RNA-dependent RNA polymerase (RdRP) to amplify the response each generation [89, 90]. In *C. elegans*, RdRPs act in a non-processive manner to produce short products [91]. Two rounds of RdRP activity would be needed to copy a tRNA fragment, and because the product of the first round would be so short, it would be unlikely that the original tRNA sequence would be recovered after these rounds of replication. It is therefore unlikely that RdRPs could copy tRNA fragments, so tRNA fragments are likely to only survive for a very small number of generations on average, which is consistent with our observations. In addition, we did not see evidence of induction of 22G-RNAs mapping to tRNA targets. It is not therefore our current view that tRNA fragment induction acts as a significant source of epimutations that is revealed upon cisplatin treatment. Nevertheless, future work using stress to induce tRNA derived fragments in *C. elegans* could reveal new mechanisms whereby this could lead to transgenerational epigenetic changes in gene expression that persist more than one or two generations.

## Conclusions

Using *C. elegans* culture under chronic exposure to cisplatin as a model system, we show that heritable epigenetic changes arising within populations under relaxed selection (epimutations) involving changes in gene expression or small non-coding RNAs are not substantially altered by genotoxic stress. However, we show that cisplatin exposure is associated with increased fluctuations in levels of small non-coding RNAs derived from tRNAs. We conclude that cisplatin-induced genotoxic stress affects mutation rate and disturbs some classes of small non-coding RNAs but does not affect the properties of epimutations. Our study provides new insights into the extent to which epimutations conform to classical models of evolution.

### Supplementary Information

**Additional file 1:** **Fig. S1.** Identification of spontaneous epimutations under relaxed selection. A. Simulation to identify optimal Z-score cut-off. X-axis shows range of Zscore cut-offs tested. Y-axis shows difference between the percentage of epimutations inherited for two or more generations compared to the predicted percentage if epimutations occurred randomly but were never inherited. B. Identification of epimutations. Epimutated loci were defined as loci with log2(read counts)(rings on plot) that were significantly greater (UP) or lower (DOWN) than that of the same locus in the pre-epimutation accumulation generation according to a Z-score cut off. Both A and B were modifed from Wilson *et al*, 202311.

**Additional file 2:** **Table S1.** Reference table for sncRNAs type identification in the tinyRNA pipeline.

**Additional file 3:** **Table S2.** Reads count number for sncRNAs mapping to tRNAs. Readcount for each small RNA mapping to a tRNA was normalized using the size factors from DESeq calculated as part of the tinyRNA pipeline. Counts for individual tRNA types were obtained by summing normalized reads mapping to the 3’ half of each tRNA type.

**Additional file 4:** **Table S3.** tRNAs 3’ halves potential target genes. To identify potential target genes, small RNAs corresponding to tRNA 3’ halves were extracted from all the lines and combined into a single file, along with the annotation indicating which tRNA the sequence was derived from. This was aligned to the cell genome allowing up to 3 mismatches. Protein-coding genes overlapping with mismatched tRNA fragments were then obtained using bedtools intersect, to identify genes that were potentially targeted by tRNAs.

**Additional file 5:** **Table S4.** Genes involved in apoptosis in *C. elegans*. The list of genes playing a role in apoptosis and cell death was obtained from Wormbase (https://wormbase.org/).

**Additional file 6:** **Fig. S2.** Additional evidence supporting the genotoxic effect of cisplatin on worms. A. Boxplot of the number of worms in control or in cisplatin high dose condition. No significant difference was observed between the two conditions (T-test, *p* val = 0.05714). Each dot represents a plate with worms (*N* = 4 plates per condition). B. Bubble plot showing ontology term enrichment of genes with genes expression changes in high dose cisplatin (*N* = 2) compared to genes without expression change. Enrichment calculated using Fisher's Exact Test with Bonferroni correction, top 10 results shown, X-axis shows log10(Odds) or enrichment. Y-axis shows ontology terms. *P*-value cut off for significance is 0.05. Supporting information can be found in the excel file: "Additional file 27".

**Additional file 7:** **Fig. S3.** Effects of cisplatin on indels. Boxplot of the number of indels arising in each generation for control lines (blue), cisplatin low dose lines (green) and cisplatin high dose lines (red). Results from each specific line within each experimental condition are represented with different symbols. Generations within each line were used as technical replicates with for C1: *N* = 9, C2: *N* = 7, L1: *N* = 6, L2: *N* = 5, H1: *N* = 6, H2: *N* = 7. Two lines per condition were used as biological replicates. Supporting data is available in the excel file: "Additional file 28".

**Additional file 8:** **Table S5.** Gene ontology enrichment result for high dose cisplatin. 279 significantly enriched functions with genes expression changes in high dose cisplatin condition compared to control were identified using DESeq2 and EnrichR.

**Additional file 9.** Data supporting Fig. 2.

**Additional file 10.** Data supporting Table 1.

**Additional file 11: Fig. S4.** Effects of cisplatin exposure on gene expression epimutations. A. Boxplot of the number of new RNA epimutations arising at each generation of the MA lines compared to the pre-mutation generation F0 and for each lineage (represented by symbols) and in each condition: control (blue), cisplatin low dose (green) and cisplatin high dose (red). Generations within each line were used as technical replicates with C1: *N* = 10, C2: *N* = 9, L1: *N* = 10, L2: *N* = 10, H1: *N* = 9, H2: *N* = 10. Two lines per condition were used as biological replicates. B. Survival curves representing the new RNA epimutations duration in each lineage: C1 (light blue), C2 (dark blue), L1 (light green), L2 (dark green), H1 (red) and H2 (pink). C. Barplot of the mean percentage of new RNA epimutations that lasted more than 1 generation compared to total epimutations arising from each generation. Data are presented by lineage: C1 (light blue), C2 (dark blue), L1 (light green), L2 (dark green), H1 (red) and H2 (pink). Means were calculated using data from each epimutation-accumulation generation within each lineage, i.e., C1: *N* = 9, C2: *N* = 8, L1: *N* = 9, L2: *N* = 9, H1: *N* = 8, H2: *N* = 9. D. Bubble plot illustrating ontology term enrichment of RNA epimutations lasting more than 1 generation in C1 (light blue), C2 (dark blue), L1 (light green), L2 (dark green), H1 (red) and H2 (pink) compared to gene without epimutation. Enrichment was calculated with χ-squared test. The top 10 results per lineage are shown. X-axis shows log10(χ) for enrichment. Y-axis shows ontology terms. All displayed ontology terms were significantly enriched. E. Bubble plot showing the distribution of lasting RNA epimutations in C1 (light blue), C2 (dark blue), L1 (light green), L2 (dark green), H1 (red) and H2 (pink). Y-axis displays the constitutive chromatin domains investigated. Active chromatin domains correspond to domain enriched in H3K36me3 mark and regulated domains to domain enriched in H3K27me3. X-axis shows the log2(Odds) of enrichment. Odds ratio and *p*-values were calculated using Fisher’s Exact Test with Bonferroni correction. *p*-value cut off for significance is 0.05. Supporting data is available in the excel file: “Additional file 29”.

**Additional file 12:** **Table S6.** Gene ontology analysis for gene expression epimutations. Gene ontology analysis results for gene expression changes across the mutation accumulation lines

**Additional file 13:** **Fig. S5.** Epimutations in genes involved in apoptosis. Boxplot of gene expression epimutations rate for each generation of the MA lines compared to the pre-mutation generation F0 and for each condition: control (blue, *N* = 19), cisplatin low dose (green, *N* = 20) and cisplatin high dose (red, *N* = 19). No significant difference was observed between conditions (Kruskal-Wallis rank-sum test, *p*-value = 0.06). Supporting data can be found in the excel file: "Additional file 30".

**Additional file 14.** Data supporting Fig. 3.

**Additional file 15:** **Fig. S6.** Effects of cisplatin exposure on sncRNAs epimutations.A. Boxplot of 22G-RNAs epimutations rate for each generation of the MAlines compared to the pre-mutation generation F0 and for each lineage: C1 (light blue, *N* = 10), C2 (dark blue, *N* = 9), L1 (light green, *N* = 9), L2 (dark green, *N* = 9), H1 (red, *N* = 9) and H2 (pink, *N* =10). B. Survival curves representing the new 22G-RNAs epimutations duration in each lineage: C1 (light blue), C2 (dark blue), L1 (light green), L2 (dark green), H1 (red) and H2 (pink). C. Boxplot of piRNAs epimutation rate for each generation of the MA lines compared to the pre-mutation generation F0 and for each lineage: C1 (light blue, *N* = 10), C2 (dark blue, *N* = 9), L1 (light green, *N* = 9), L2 (dark green, *N* = 9), H1 (red, *N* = 9) and H2 (pink, *N* =10). D. Survival curves representing the new piRNAs epimutations duration in each exposure lineage: C1 (light blue), C2 (dark blue), L1 (light green), L2 (dark green), H1 (red) and H2 (pink). E. Boxplot of miRNAs epimutations rate for each generation of the MA lines compared to the pre-mutation generation F0 and for each lineage: C1 (light blue, *N* = 10), C2 (dark blue, *N* = 9), L1 (light green, *N* = 9), L2 (dark green, *N* = 9), H1 (red, *N* = 9) and H2 (pink, *N* =10). F. Survival curves representing the new miRNAs epimutations duration in each exposure lineage: C1 (light blue), C2 (dark blue), L1 (light green), L2 (dark green), H1 (red) and H2 (pink). G. Boxplot of 26G-RNAs epimutations rate for each generation of the MA lines compared to the pre-mutation generation F0 and for each lineage: C1 (light blue, *N* = 10), C2 (dark blue, *N* = 9), L1 (light green, *N* = 9), L2 (dark green, *N* = 9), H1 (red, *N* = 9) and H2 (pink, *N* =10). H. Survival curves representing the new 26G-RNAs epimutations duration in each exposure lineage: C1 (light blue), C2 (dark blue), L1 (light green), L2 (dark green), H1 (red) and H2 (pink). Supporting information is available in the excel file: "Additional file 31".

**Additional file 16:** **Fig. S7.** Association between 22G-RNAs epimutations and gene expression epimutations. A. Stacked barplots showing the percentage of genes with inherited (left-bar) of non-inherited (right-bar) RNA expression changes targeted by 22G-RNAs epimutations in control condition (left panel), cisplatin low dose condition (middle panel) and cisplatin high dose condition (right panel). In blue the percentage of genes with simultaneous inherited 22G-RNAs epimutations, in grey the percentage of genes with simultaneous non-inherited 22G-RNAs epimutations and in orange the percentage of genes with non-simultaneous 22G-RNAs epimutations. For each condition, data from two lineages were combined. Supporting data can be found in the excel file: "Additional file 32"

**Additional file 17:** **Table S7.** Stepwise analysis of association of gene expression changes and 22G-RNAs epimutations in the different exposure conditions. To test the association between 22G-RNAs epimutations and gene expression epimutations, we first looked at all genes as a background regardless of whether they had expression change or not. Then, the proportion of genes with gene expression epimutation was compared to the proportion of genes which had 22G-RNAs epimutation. The overlap, in which genes had both expression changes and 22G-RNA epimutations, was calculated (Test 1). In a second time, only genes with gene expression epimutation were selected and among these genes, the proportion of genes with inherited expression changes was compared with the proportion of genes with 22G-RNA epimutations and the overlap was calculated (Test 2). In third, among genes with genes expression changes, the proportion of ones with inherited expression change was compared with the proportion of genes with simultaneous 22G-RNA epimutation. The overlap of genes with both conditions was calculated (Test 3) Finally, this third analysis was repeated but among genes with BOTH gene expression change and 22G-RNA epimutation (Test 4). Odds ratios and *p*-values for each test and in each condition were calculated with Fisher’s Exact Test. Cells in yellow indicate significantly increased odds of association, cells in grey indicate no significant association.

**Additional file 18.** Data supporting Fig. 4.

**Additional file 19:** **Fig. S8.** Cisplatin effects on tRNAs. A. Total normalized reads that mapped to tRNAs in each lineage: control 1 (blue, *N* = 11), control 2 (light blue, *N* = 10), cisplatin low dose 1 (green, *N* = 10), cisplatin low dose 2 (dark green, *N* = 10), cisplatin high dose 1 (red, *N* = 10) and cisplatin high dose 2 (dark red, *N* = 11). B. Total normalized reads represented according to their mapping positions on the respective tRNA sequence and in each different lineage: control 1 (blue, *N* = 11), control 2 (light blue, *N* = 10), cisplatin low dose 1 (green, *N* = 10), cisplatin low dose 2 (dark green, *N* = 10), cisplatin high dose 1 (red, *N* = 10) and cisplatin high dose 2 (dark red, *N* = 11). C. Violin plot of total normalized tRNAs mapping specifically to the 3’ half of the tRNAs in the different lineage: control 1 (blue, *N* = 11), control 2 (light blue, *N* = 10), cisplatin low dose 1 (green, *N* = 10), cisplatin low dose 2 (dark green, *N* = 10), cisplatin high dose 1 (red, *N* = 10) and cisplatin high dose 2 (dark red, *N* = 11). D. Boxplot of the number of new epimutations affecting tRNAs fragments arising at each generation of the MA lines compared to the pre-mutation generation F0 and for each lineage: control 1 (blue, *N* = 10), control 2 (light blue, *N* = 9), cisplatin low dose 1 (green, *N* = 9), cisplatin low dose 2 (dark green, *N* = 9), cisplatin high dose 1 (red, *N* = 9) and cisplatin high dose 2 (dark red, *N* = 10). Supporting data is provided in the excel file: "Additional file 33".

**Additional file 20:** **Fig. S9.** Association between tRNAs and specific AGOs proteins. A. Plot of total tRNAs count (in reads per million) in wild type C. elegans (N2; black diamonds) and mutants lacking specific AGOs proteins (from left to right: ergo1 (blue diamonds), rde1 (yellow diamonds) and wago10 (red diamonds). A very small decrease in wago10 was observed. *N* = 3 per mutant. B. Plot representing specifically Glutamate tRNA (GluCTC (diamonds) and GluTCC (dots) fragments (in reads per million) in wild type C. elegans (N2; black) and in mutants lacking wago10 (red). *N* = 5 for N2 with 2 replicates for GluCTC and 3 for GluTCC. *N* = 6 for wago10 mutant with 3 replicates for both tRNAs. A significant difference in Glutamate tRNA fragments was observed between wild type worms and mutants lacking wago10 (Wilcoxon-test, adjusted *p*-value = 0.05). Supporting data are available in the excel file: "Additional file 34".

**Additional file 21:** **Table S8.** Number and maximal duration of epimutations by tRNAs type.

**Additional file 22:** **Fig. S10.** Detailed characterisation of tRNAs epimutations. A. Barplot of total number of epimutations for each tRNAs type and in the different conditions: control (blue), LD (green) and HD (red). B. Barplot of the duration of tRNAs epimutations for each kind and according to the exposure condition: control (blue), LD (green) and HD (red). C. Forest plot of Cox Proportional Hazards Model representing the odd of difference in the tRNAs 3’ halves epimutations between the conditions. The x-axis show the chances of an epimutation to disappear in the cisplatin conditions in comparison to control (reference). The *p*-values were calculated using log rank test. For A, B and C, two lineages for each condition were used as biological replicates. Supporting data can be found in the excel file: "Additional file 35".

**Additional file 23.** Data supporting Fig. 5.

**Additional file 24:** **Table S9.** Table of tRNAs 3’ halves epimutations associated with apoptotic genes. Only the gene vps-16 was identified as a potential tRNAs target. No associated tRNA 3’ halve epimutation was observed and thus for all lineages and in all conditions.

**Additional file 25.** Data supporting Fig. 6.

**Additional file 26.** Data supporting Fig. 7.

**Additional file 27.** Data supporting Fig. S2.

**Additional file 28.** Data supporting Fig. S3.

**Additional file 29.** Data supporting Fig. S4.

**Additional file 30.** Data supporting Fig. S5.

**Additional file 31.** Data supporting Fig. S6.

**Additional file 32.** Data supporting Fig. S7.

**Additional file 33.** Data supporting Fig. S8.

**Additional file 34.** Data supporting Fig. S9.

**Additional file 35.** Data supporting Fig. S10.

## Data Availability

All data generated or analysed during this study are included in the published article, its supplementary information files, and publicly available datasets. The sncRNA data have been deposited with links to BioProject accession number PRJNA991138 in the NCBI BioProject database (https://www.ncbi.nlm.nih.gov/bioproject/). All code for the R analysis including figure plotting commands can be found on Zenodo at the following https://doi.org/10.5281/zenodo.10091768.

## Abbreviations

RISC: RNA‐induced silencing complex
AGO: Argonaute protein
WAGO: Worm-specific Argonaute protein
MA: Mutation accumulation
NGM: Nematode growth medium
RppH: RNA pyroxophosphohydrolase
ATACseq Assay: Transposase accessible chromatin sequencing
LD: Low-dose cisplatin
HD: High-dose cisplatin
WT: Wild type
DE: Differentially expressed
sncRNA: Small non-coding RNA
piRNA: Piwi-interacting small RNA
miRNA: MicroRNA
