# Supplementary material for: Cisplatin exposure alters tRNA-derived small RNAs but does not affect epimutations in C. elegans
Source: BMC Biol. 2023 Nov 29;21:276. doi: 10.1186/s12915-023-01767-z (PMC10688063; doi:10.1186/s12915-023-01767-z)
Supplement: Supplementary file 1 — Additional file 1: Fig. S1. Identification of spontaneous epimutations under relaxed selection. A. Simulation to identify optimal Z-score cut-off. X-axis shows range of Zscore cut-offs tested. Y-axis shows difference between the percentage of epimutations inherited for two or more generations compared to the predicted percentage if epimutations occurred randomly but were never inherited. B. Identification of epimutations. Epimutated loci were defined as loci with log2(read counts)(rings on plot) that were significantly greater (UP) or lower (DOWN) than that of the same locus in the pre-epimutation accumulation generation according to a Z-score cut off. Both A and B were modifed from Wilson et al, 202311. [file 12915_2023_1767_MOESM1_ESM.pdf]

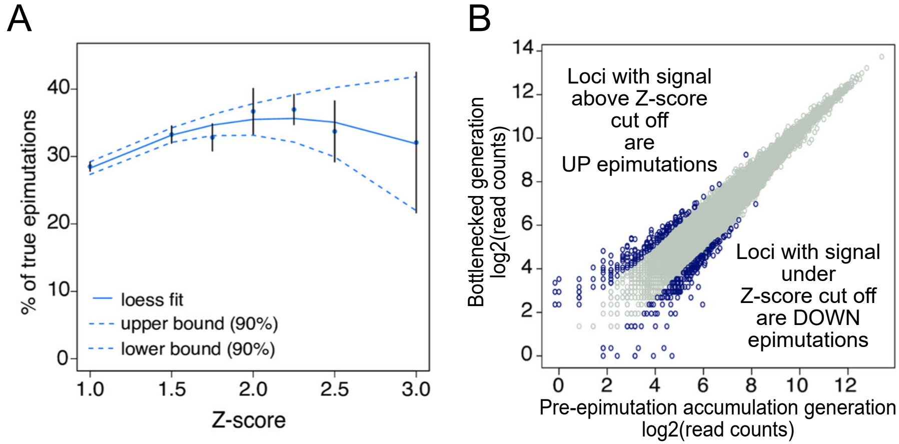

Fig. S1: Identification of spontaneous epimutations under relaxed selection. A. Simulation to identify optimal Z-score cut-off. X-axis shows range of Zscore cut-offs tested. Y-axis shows difference between the percentage of epimutations inherited for two or more generations compared to the predicted percentage if epimutations occurred randomly but were never inherited. B. Identification of epimutations. Epimutated loci were defined as loci with log2(read counts)(rings on plot) that were significantly greater (UP) or lower (DOWN) than that of the same locus in the pre-epimutation accumulation generation according to a Z-score cut off. Both A and B were modifed from Wilson *et al*, 2023[11].

Figure S1
