# Supplementary material for: Cisplatin exposure alters tRNA-derived small RNAs but does not affect epimutations in C. elegans
Source: BMC Biol. 2023 Nov 29;21:276. doi: 10.1186/s12915-023-01767-z (PMC10688063; doi:10.1186/s12915-023-01767-z)
Supplement: Supplementary file 6 — Additional file 6: Fig. S2. Additional evidence supporting the genotoxic effect of cisplatin on worms. A. Boxplot of the number of worms in control or in cisplatin high dose condition. No significant difference was observed between the two conditions (T-test, p val = 0.05714). Each dot represents a plate with worms (N = 4 plates per condition). B. Bubble plot showing ontology term enrichment of genes with genes expression changes in high dose cisplatin (N = 2) compared to genes without expression change. Enrichment calculated using Fisher's Exact Test with Bonferroni correction, top 10 results shown, X-axis shows log10(Odds) or enrichment. Y-axis shows ontology terms. P-value cut off for significance is 0.05. Supporting information can be found in the excel file: "Additional file 27". [file 12915_2023_1767_MOESM6_ESM.pdf]

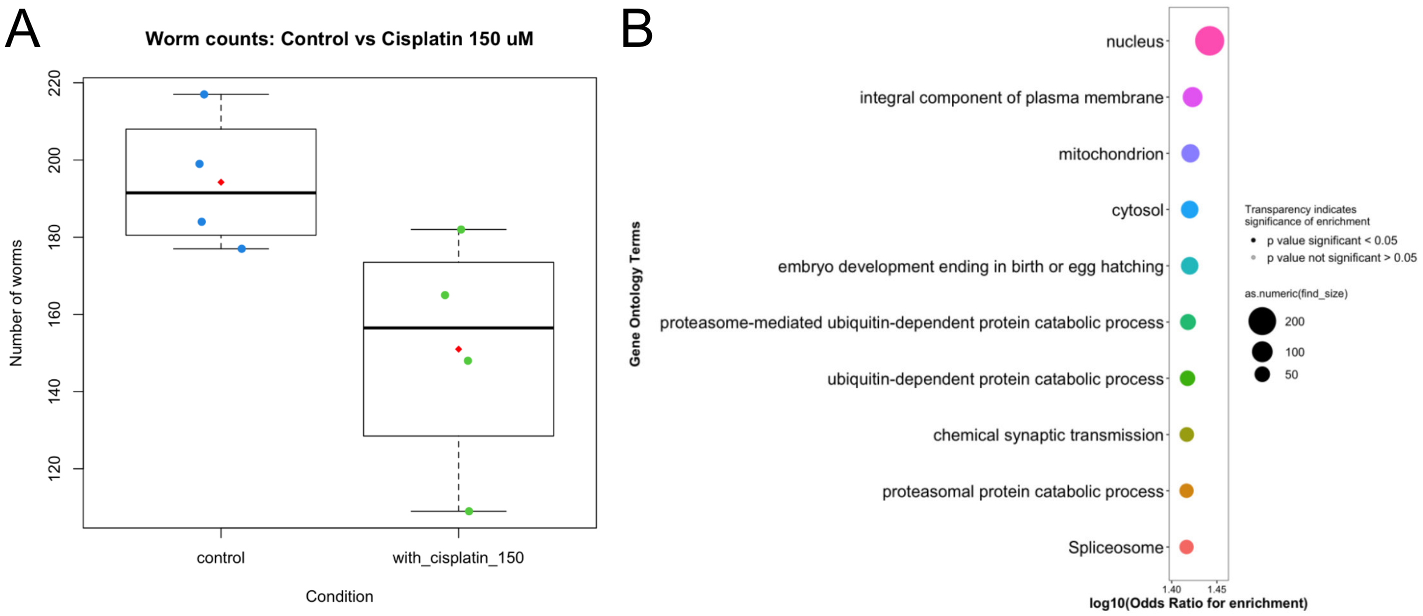

Fig. S2: Additional evidences supporting the genotoxic effect of cisplatin on worms. A. Boxplot of the number of worms in control or in cisplatin high dose condition. No significant difference was observed between the two conditions (T-test, pval=0.05714). Each dot represents a plate with worms (N = 4 plates per condition). B. Bubble plot showing ontology term enrichment of genes with genes expression changes in high dose cisplatin (N = 2) compared to genes without expression change. Enrichment calculated using Fisher's Exact Test with Bonferroni correction, top 10 results shown, X-axis shows log10(Odds) or enrichment. Y-axis shows ontology terms. P-value cut off for significance is 0.05.
Supporting information can be found in the excel file: "Additional file 27".

Figure S2
