# Supplementary material for: Cisplatin exposure alters tRNA-derived small RNAs but does not affect epimutations in C. elegans
Source: BMC Biol. 2023 Nov 29;21:276. doi: 10.1186/s12915-023-01767-z (PMC10688063; doi:10.1186/s12915-023-01767-z)
Supplement: Supplementary file 7 — Additional file 7: Fig. S3. Effects of cisplatin on indels. Boxplot of the number of indels arising in each generation for control lines (blue), cisplatin low dose lines (green) and cisplatin high dose lines (red). Results from each specific line within each experimental condition are represented with different symbols. Generations within each line were used as technical replicates with for C1: N = 9, C2: N = 7, L1: N = 6, L2: N = 5, H1: N = 6, H2: N = 7. Two lines per condition were used as biological replicates. Supporting data is available in the excel file: "Additional file 28". [file 12915_2023_1767_MOESM7_ESM.pdf]

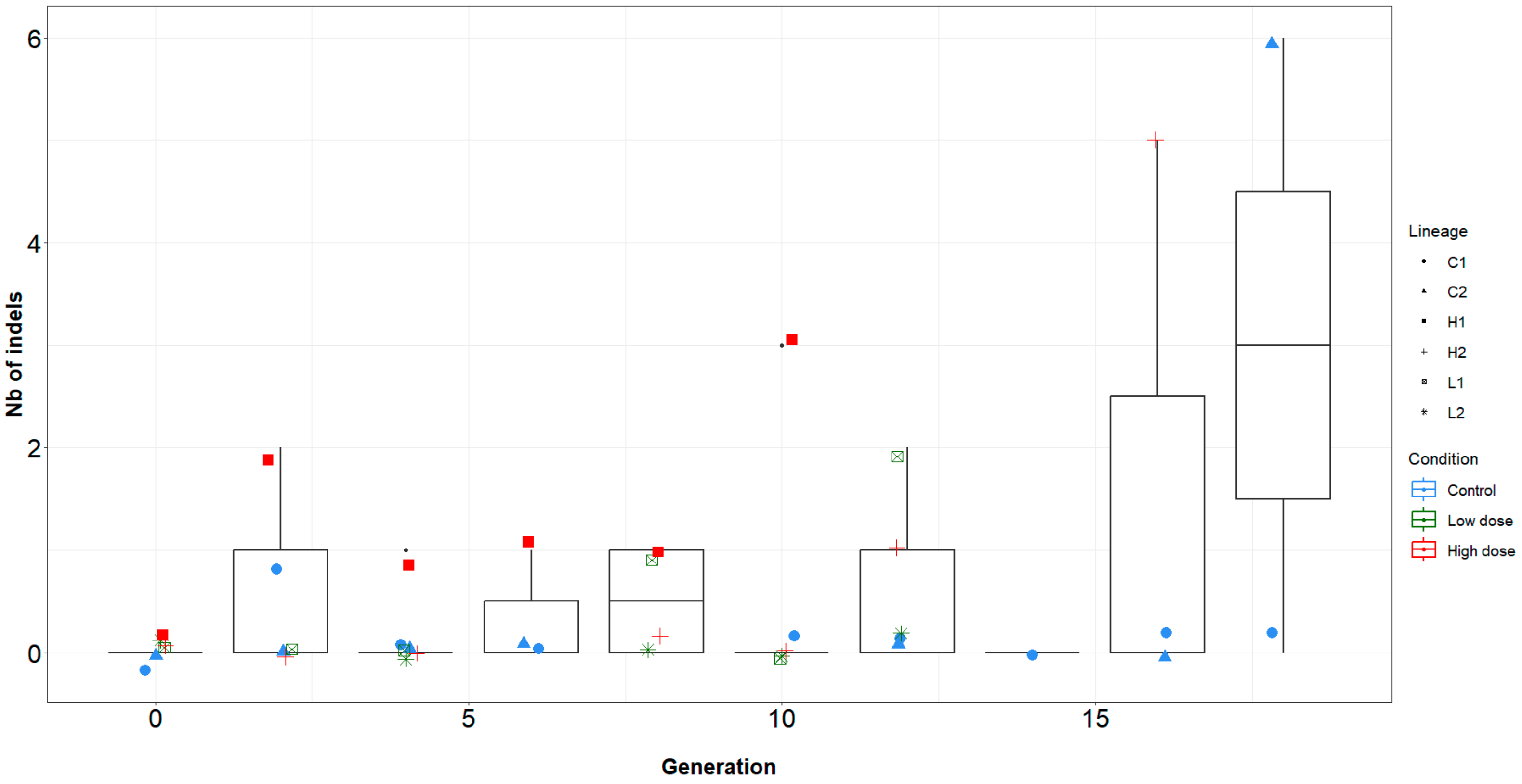

Fig. S3: Effects of cisplatin on gene expression epimutations. Boxplot of the number of indels arising in each generation for control lines (blue), cisplatin low dose lines (green) and cisplatin high dose lines (red). Results from each specific line within each experimental condition are represented with different symbols. Generations within each line were used as technical replicates with for C1: N = 9, C2: N = 7, L1: N = 6, L2: N = 5, H1: N = 6, H2: N = 7. Two lines per condition were used as biological replicates. Supporting data is available in the excel file: "Additional file 28".

Figure S3
