# Supplementary material for: Cisplatin exposure alters tRNA-derived small RNAs but does not affect epimutations in C. elegans
Source: BMC Biol. 2023 Nov 29;21:276. doi: 10.1186/s12915-023-01767-z (PMC10688063; doi:10.1186/s12915-023-01767-z)
Supplement: Supplementary file 11 — Additional file 11: Fig. S4. Effects of cisplatin exposure on gene expression epimutations. A. Boxplot of the number of new RNA epimutations arising at each generation of the MA lines compared to the pre-mutation generation F0 and for each lineage (represented by symbols) and in each condition: control (blue), cisplatin low dose (green) and cisplatin high dose (red). Generations within each line were used as technical replicates with C1: N = 10, C2: N = 9, L1: N = 10, L2: N = 10, H1: N = 9, H2: N = 10. Two lines per condition were used as biological replicates. B. Survival curves representing the new RNA epimutations duration in each lineage: C1 (light blue), C2 (dark blue), L1 (light green), L2 (dark green), H1 (red) and H2 (pink). C. Barplot of the mean percentage of new RNA epimutations that lasted more than 1 generation compared to total epimutations arising from each generation. Data are presented by lineage: C1 (light blue), C2 (dark blue), L1 (light green), L2 (dark green), H1 (red) and H2 (pink). Means were calculated using data from each epimutation-accumulation generation within each lineage, i.e., C1: N = 9, C2: N = 8, L1: N = 9, L2: N = 9, H1: N = 8, H2: N = 9. D. Bubble plot illustrating ontology term enrichment of RNA epimutations lasting more than 1 generation in C1 (light blue), C2 (dark blue), L1 (light green), L2 (dark green), H1 (red) and H2 (pink) compared to gene without epimutation. Enrichment was calculated with χ-squared test. The top 10 results per lineage are shown. X-axis shows log10(χ) for enrichment. Y-axis shows ontology terms. All displayed ontology terms were significantly enriched. E. Bubble plot showing the distribution of lasting RNA epimutations in C1 (light blue), C2 (dark blue), L1 (light green), L2 (dark green), H1 (red) and H2 (pink). Y-axis displays the constitutive chromatin domains investigated. Active chromatin domains correspond to domain enriched in H3K36me3 mark and regulated domains to domain enriched in H3K27me3. X-ax [file 12915_2023_1767_MOESM11_ESM.pdf]

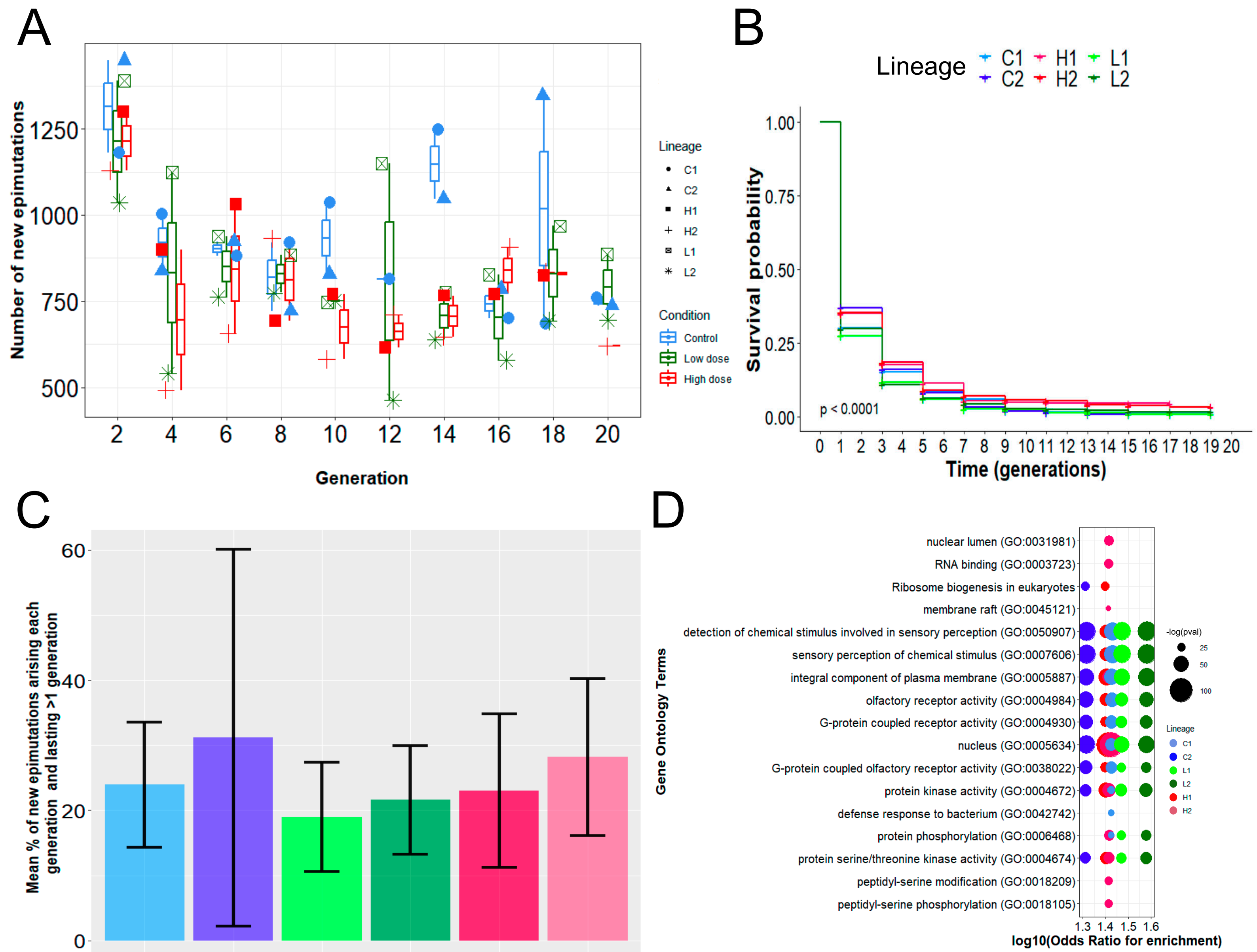

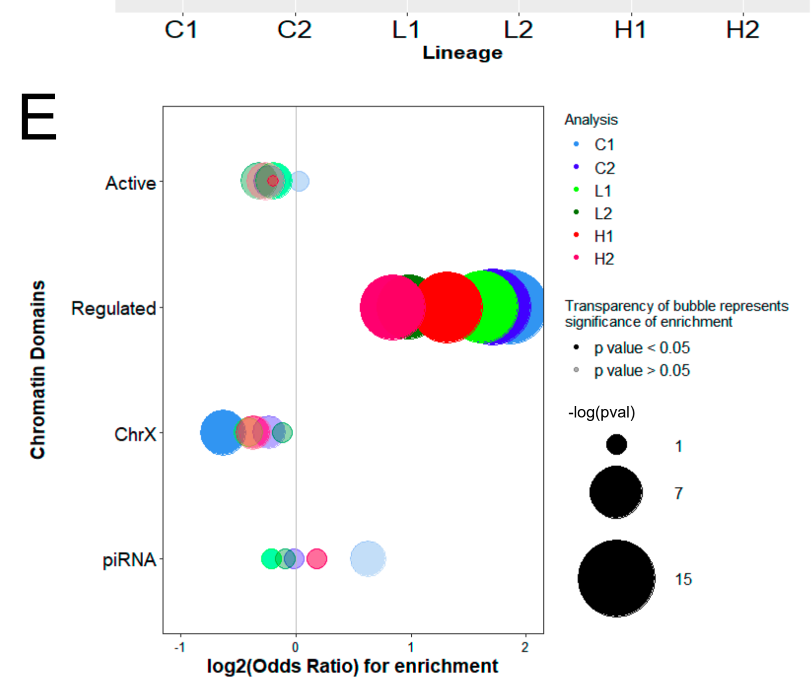

Fig. S4: Effects of cisplatin exposure on gene expression epimutations. A. Boxplot of the number of new RNA epimutations arising at each generation of the MA lines compared to the pre-mutation generation F0 and for each lineage (represented by symbols) and in each condition: control (blue), cisplatin low dose (green) and cisplatin high dose (red). Generations within each line were used as technical replicates with C1: N = 10, C2: N = 9, L1: N = 10, L2: N = 10, H1: N = 9, H2: N = 10. Two lines per condition were used as biological replicates. B. Survival curves representing the new RNA epimutations duration in each lineage: C1 (light blue), C2 (dark blue), L1 (light green), L2 (dark green), H1 (red) and H2 (pink). C. Barplot of the mean percentage of new RNA epimutations that lasted more than 1 generation compared to total epimutations arising from each generation. Data are presented by lineage: C1 (light blue), C2 (dark blue), L1 (light green), L2 (dark green), H1 (red) and H2 (pink). Means were calculated using data from each epimutation-accumulation generation within each lineage, i.e. C1: N = 9, C2: N = 8, L1: N = 9, L2: N = 9, H1: N = 8, H2: N = 9. D. Bubble plot illustrating ontology term enrichment of RNA epimutations lasting more than 1 generation in C1 (light blue), C2 (dark blue), L1 (light green), L2 (dark green), H1 (red) and H2 (pink) compared to gene without epimutation. Enrichment was calculated with χ-squared test. The top 10 results per lineage are shown. X-axis shows log10(χ) for enrichment. Y-axis shows ontology terms. All displayed ontology terms were significantly enriched. E. Bubble plot showing the distribution of lasting RNA epimutations in C1 (light blue), C2 (dark blue), L1 (light green), L2 (dark green), H1 (red) and H2 (pink). Y-axis displays the constitutive chromatin domains investigated. Active chromatin domains correspond to domain enriched in H3K36me3 mark and regulated domains to domain enriched in H3K27me3. X-axis shows the log2(Odds) of enrichment. Odds ratio and p-values were calculated using Fisher's Exact Test with Bonferroni correction. p-value cut off for significance is 0.05. Supporting data is available in the excel file: "Additional file 29".

Figure S4
