# Supplementary figures and images for: Cisplatin exposure alters tRNA-derived small RNAs but does not affect epimutations in C. elegans
Source: BMC Biol. 2023 Nov 29;21:276. doi: 10.1186/s12915-023-01767-z (PMC10688063; doi:10.1186/s12915-023-01767-z)

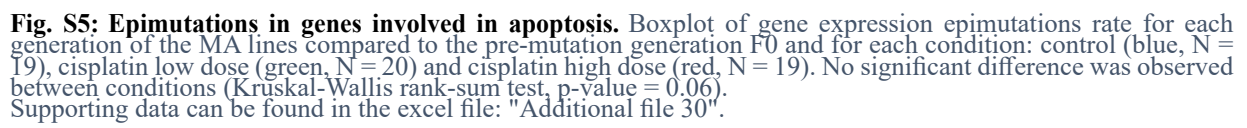

Supplement: Supplementary file 13 — Additional file 13: Fig. S5. Epimutations in genes involved in apoptosis. Boxplot of gene expression epimutations rate for each generation of the MA lines compared to the pre-mutation generation F0 and for each condition: control (blue, N = 19), cisplatin low dose (green, N = 20) and cisplatin high dose (red, N = 19). No significant difference was observed between conditions (Kruskal-Wallis rank-sum test, p-value = 0.06). Supporting data can be found in the excel file: "Additional file 30". [file 12915_2023_1767_MOESM13_ESM.pdf]
