# Supplementary material for: Cisplatin exposure alters tRNA-derived small RNAs but does not affect epimutations in C. elegans
Source: BMC Biol. 2023 Nov 29;21:276. doi: 10.1186/s12915-023-01767-z (PMC10688063; doi:10.1186/s12915-023-01767-z)
Supplement: Supplementary file 15 — Additional file 15: Fig. S6. Effects of cisplatin exposure on sncRNAs epimutations.A. Boxplot of 22G-RNAs epimutations rate for each generation of the MAlines compared to the pre-mutation generation F0 and for each lineage: C1 (light blue, N = 10), C2 (dark blue, N = 9), L1 (light green, N = 9), L2 (dark green, N = 9), H1 (red, N = 9) and H2 (pink, N =10). B. Survival curves representing the new 22G-RNAs epimutations duration in each lineage: C1 (light blue), C2 (dark blue), L1 (light green), L2 (dark green), H1 (red) and H2 (pink). C. Boxplot of piRNAs epimutation rate for each generation of the MA lines compared to the pre-mutation generation F0 and for each lineage: C1 (light blue, N = 10), C2 (dark blue, N = 9), L1 (light green, N = 9), L2 (dark green, N = 9), H1 (red, N = 9) and H2 (pink, N =10). D. Survival curves representing the new piRNAs epimutations duration in each exposure lineage: C1 (light blue), C2 (dark blue), L1 (light green), L2 (dark green), H1 (red) and H2 (pink). E. Boxplot of miRNAs epimutations rate for each generation of the MA lines compared to the pre-mutation generation F0 and for each lineage: C1 (light blue, N = 10), C2 (dark blue, N = 9), L1 (light green, N = 9), L2 (dark green, N = 9), H1 (red, N = 9) and H2 (pink, N =10). F. Survival curves representing the new miRNAs epimutations duration in each exposure lineage: C1 (light blue), C2 (dark blue), L1 (light green), L2 (dark green), H1 (red) and H2 (pink). G. Boxplot of 26G-RNAs epimutations rate for each generation of the MA lines compared to the pre-mutation generation F0 and for each lineage: C1 (light blue, N = 10), C2 (dark blue, N = 9), L1 (light green, N = 9), L2 (dark green, N = 9), H1 (red, N = 9) and H2 (pink, N =10). H. Survival curves representing the new 26G-RNAs epimutations duration in each exposure lineage: C1 (light blue), C2 (dark blue), L1 (light green), L2 (dark green), H1 (red) and H2 (pink). Supporting information is available in the excel file: "Additional file [file 12915_2023_1767_MOESM15_ESM.pdf]

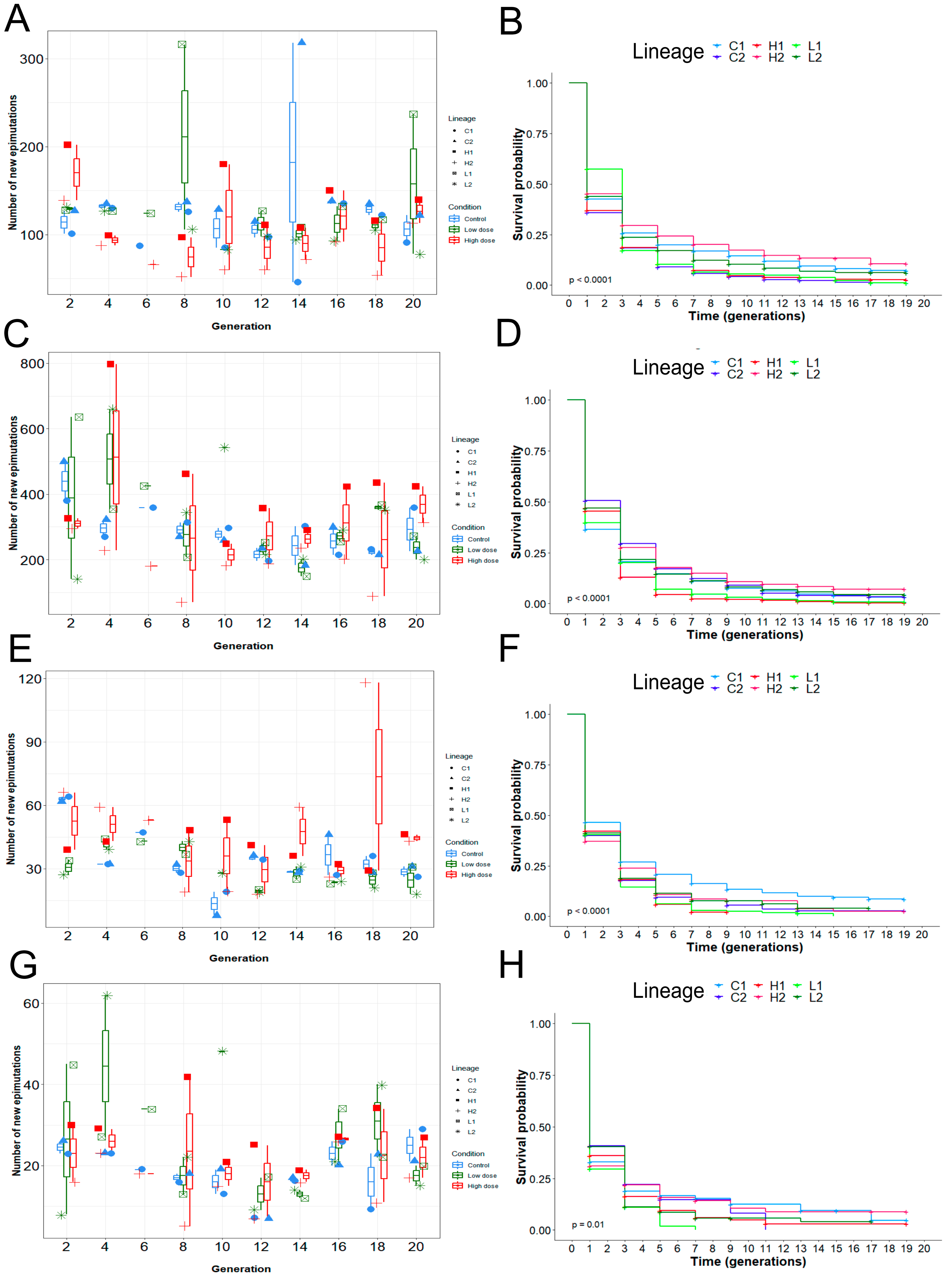

A
B
C
D
E
F
G
H
Number of new epimutations
Generation
Lineage
C1
C2
H1
H2
L1
L2
Condition
Control
Low dose
High dose
Survival probability
Time (generations)
p < 0.0001
p = 0.01

Figure S6

Fig. S6: Effects of cisplatin exposure on sncRNAs epimutations. A. Boxplot of 22G-RNAs epimutations rate for each generation of the MA lines compared to the pre-mutation generation F0 and for each lineage: C1 (light blue, N = 10), C2 (dark blue, N = 9), L1 (light green, N = 9), L2 (dark green, N = 9), H1 (red, N = 9) and H2 (pink, N =10). B. Survival curves representing the new 22G-RNAs epimutations duration in each lineage: C1 (light blue), C2 (dark blue), L1 (light green), L2 (dark green), H1 (red) and H2 (pink). C. Boxplot of piRNAs epimutation rate for each generation of the MA lines compared to the pre-mutation generation F0 and for each lineage: C1 (light blue, N = 10), C2 (dark blue, N = 9), L1 (light green, N = 9), L2 (dark green, N = 9), H1 (red, N = 9) and H2 (pink, N =10). D. Survival curves representing the new piRNAs epimutations duration in each exposure lineage: C1 (light blue), C2 (dark blue), L1 (light green), L2 (dark green), H1 (red) and H2 (pink). E. Boxplot of miRNAs epimutations rate for each generation of the MA lines compared to the pre-mutation generation F0 and for each lineage: C1 (light blue, N = 10), C2 (dark blue, N = 9), L1 (light green, N = 9), L2 (dark green, N = 9), H1 (red, N = 9) and H2 (pink, N =10). F. Survival curves representing the new miRNAs epimutations duration in each exposure lineage: C1 (light blue), C2 (dark blue), L1 (light green), L2 (dark green), H1 (red) and H2 (pink). G. Boxplot of 26G-RNAs epimutations rate for each generation of the MA lines compared to the pre-mutation generation F0 and for each lineage: C1 (light blue, N = 10), C2 (dark blue, N = 9), L1 (light green, N = 9), L2 (dark green, N = 9), H1 (red, N = 9) and H2 (pink, N =10). H. Survival curves representing the new 26G-RNAs epimutations duration in each exposure lineage: C1 (light blue), C2 (dark blue), L1 (light green), L2 (dark green), H1 (red) and H2 (pink).
Supporting information is available in the excel file: "Additional file 31".

Figure S6
