# Supplementary material for: Cisplatin exposure alters tRNA-derived small RNAs but does not affect epimutations in C. elegans
Source: BMC Biol. 2023 Nov 29;21:276. doi: 10.1186/s12915-023-01767-z (PMC10688063; doi:10.1186/s12915-023-01767-z)
Supplement: Supplementary file 16 — Additional file 16: Fig. S7. Association between 22G-RNAs epimutations and gene expression epimutations. A. Stacked barplots showing the percentage of genes with inherited (left-bar) of non-inherited (right-bar) RNA expression changes targeted by 22G-RNAs epimutations in control condition (left panel), cisplatin low dose condition (middle panel) and cisplatin high dose condition (right panel). In blue the percentage of genes with simultaneous inherited 22G-RNAs epimutations, in grey the percentage of genes with simultaneous non-inherited 22G-RNAs epimutations and in orange the percentage of genes with non-simultaneous 22G-RNAs epimutations. For each condition, data from two lineages were combined. Supporting data can be found in the excel file: "Additional file 32" [file 12915_2023_1767_MOESM16_ESM.pdf]

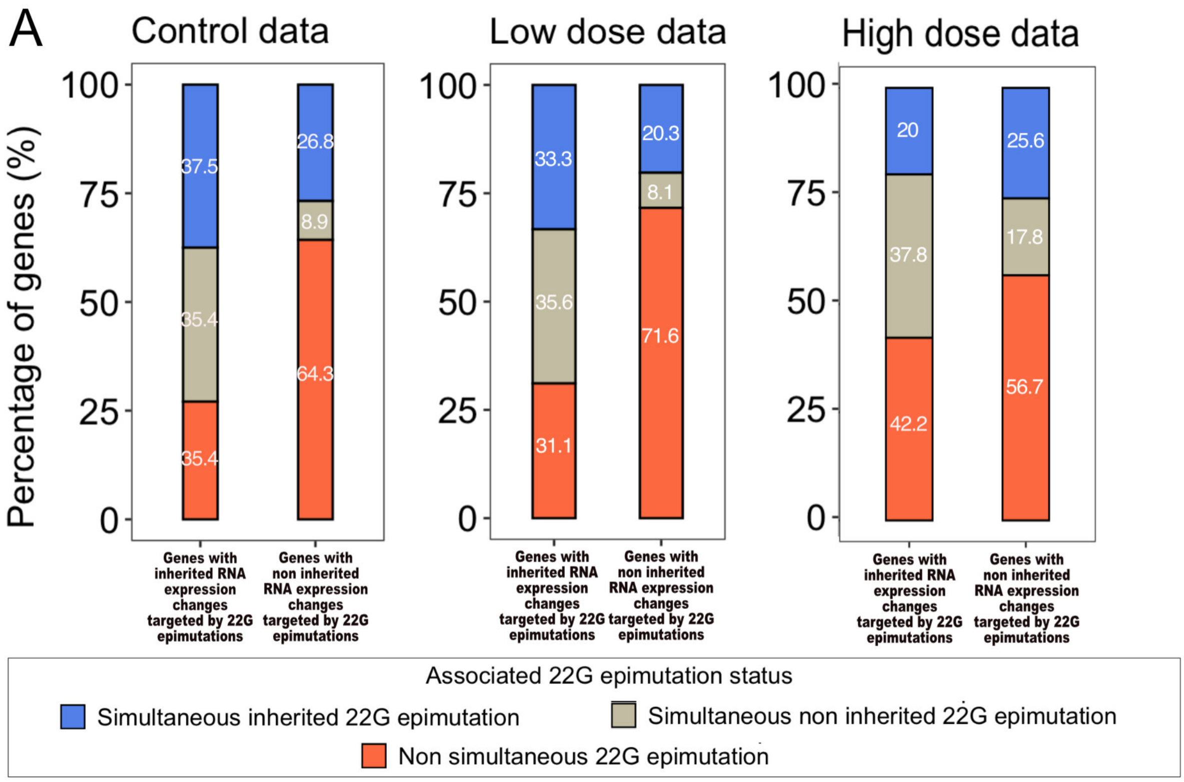

Fig. S7: Association between 22G-RNAs epimutations and gene expression epimutations. A. Stacked barplots showing the percentage of genes with inherited (left-bar) of non-inherited (right-bar) RNA expression changes targeted by 22G-RNAs epimutations in control condition (left panel), cisplatin low dose condition (middle panel) and cisplatin high dose condition (right panel). In blue the percentage of genes with simultaneous inherited 22G-RNAs epimutations, in grey the percentage of genes with simultaneous non-inherited 22G-RNAs epimutations and in orange the percentage of genes with non-simultaneous 22G-RNAs epimutations. For each condition, data from two lineages were combined.
Supporting data can be found in the excel file: "Additional file 32".

Figure S7
