# Supplementary material for: Cisplatin exposure alters tRNA-derived small RNAs but does not affect epimutations in C. elegans
Source: BMC Biol. 2023 Nov 29;21:276. doi: 10.1186/s12915-023-01767-z (PMC10688063; doi:10.1186/s12915-023-01767-z)
Supplement: Supplementary file 19 — Additional file 19: Fig. S8. Cisplatin effects on tRNAs. A. Total normalized reads that mapped to tRNAs in each lineage: control 1 (blue, N = 11), control 2 (light blue, N = 10), cisplatin low dose 1 (green, N = 10), cisplatin low dose 2 (dark green, N = 10), cisplatin high dose 1 (red, N = 10) and cisplatin high dose 2 (dark red, N = 11). B. Total normalized reads represented according to their mapping positions on the respective tRNA sequence and in each different lineage: control 1 (blue, N = 11), control 2 (light blue, N = 10), cisplatin low dose 1 (green, N = 10), cisplatin low dose 2 (dark green, N = 10), cisplatin high dose 1 (red, N = 10) and cisplatin high dose 2 (dark red, N = 11). C. Violin plot of total normalized tRNAs mapping specifically to the 3’ half of the tRNAs in the different lineage: control 1 (blue, N = 11), control 2 (light blue, N = 10), cisplatin low dose 1 (green, N = 10), cisplatin low dose 2 (dark green, N = 10), cisplatin high dose 1 (red, N = 10) and cisplatin high dose 2 (dark red, N = 11). D. Boxplot of the number of new epimutations affecting tRNAs fragments arising at each generation of the MA lines compared to the pre-mutation generation F0 and for each lineage: control 1 (blue, N = 10), control 2 (light blue, N = 9), cisplatin low dose 1 (green, N = 9), cisplatin low dose 2 (dark green, N = 9), cisplatin high dose 1 (red, N = 9) and cisplatin high dose 2 (dark red, N = 10). Supporting data is provided in the excel file: "Additional file 33". [file 12915_2023_1767_MOESM19_ESM.pdf]

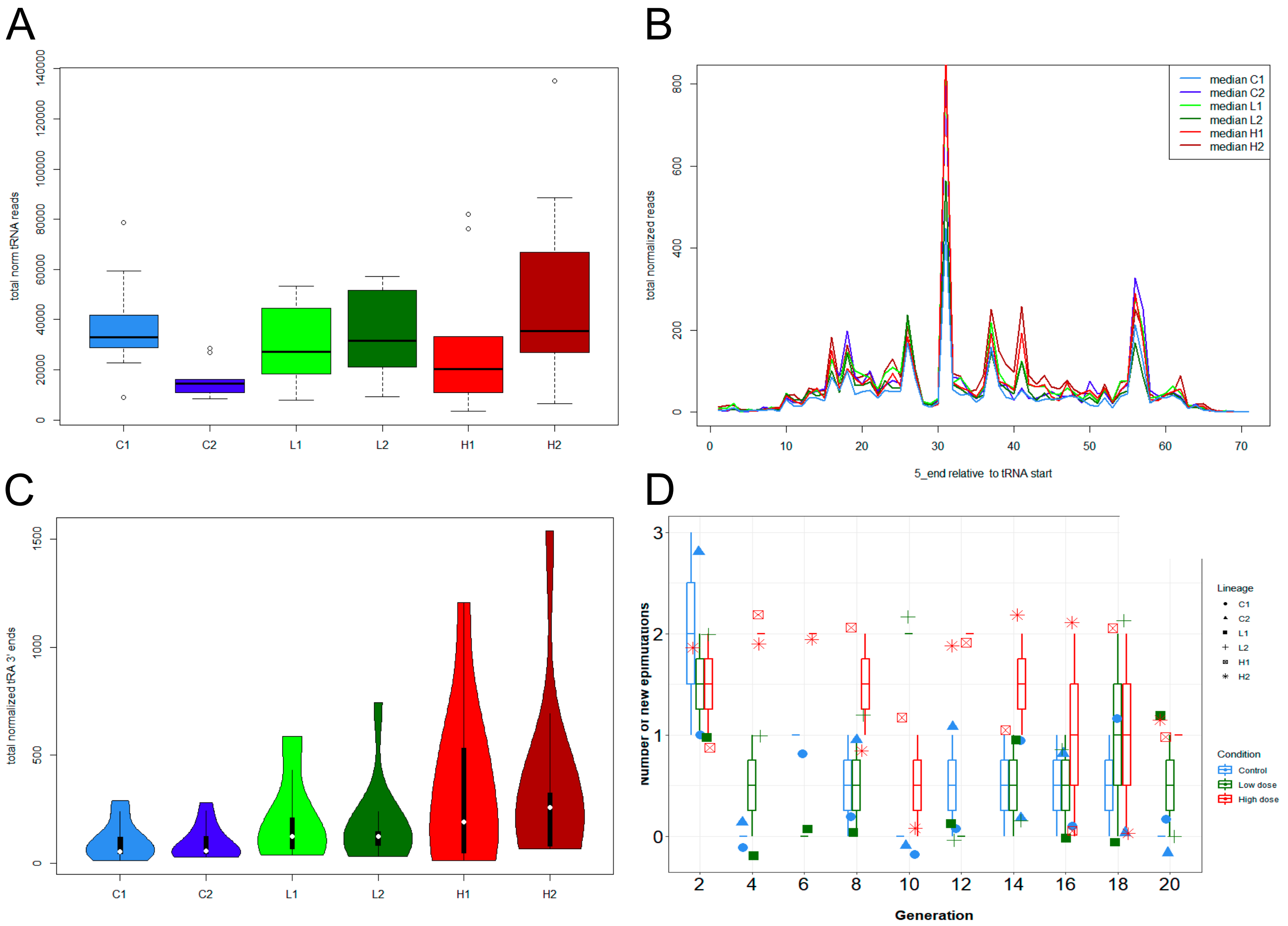

Fig. S8: Cisplatin effects on tRNAs. A. Total normalized reads that mapped to tRNAs in each lineage: control 1 (blue, N = 11), control 2 (light blue, N = 10), cisplatin low dose 1 (green, N = 10), cisplatin low dose 2 (dark green, N = 10), cisplatin high dose 1 (red, N = 10) and cisplatin high dose 2 (dark red, N = 11). B. Total normalized reads represented according to their mapping positions on the respective tRNA sequence and in each different lineage: control 1 (blue, N = 11), control 2 (light blue, N = 10), cisplatin low dose 1 (green, N = 10), cisplatin low dose 2 (dark green, N = 10), cisplatin high dose 1 (red, N = 10) and cisplatin high dose 2 (dark red, N = 11). C. Violin plot of total normalized tRNAs mapping specifically to the 3' half of the tRNAs in the different lineage: control 1 (blue, N = 11), control 2 (light blue, N = 10), cisplatin low dose 1 (green, N = 10), cisplatin low dose 2 (dark green, N = 10), cisplatin high dose 1 (red, N = 10) and cisplatin high dose 2 (dark red, N = 11). D. Boxplot of the number of new epimutations affecting tRNAs fragments arising at each generation of the MA lines compared to the pre-mutation generation F0 and for each lineage: control 1 (blue, N = 10), control 2 (light blue, N = 9), cisplatin low dose 1 (green, N = 9), cisplatin low dose 2 (dark green, N = 9), cisplatin high dose 1 (red, N = 9) and cisplatin high dose 2 (dark red, N = 10).
Supporting data is provided in the excel file: "Additional file 33".

Figure S8
