# Supplementary material for: Cisplatin exposure alters tRNA-derived small RNAs but does not affect epimutations in C. elegans
Source: BMC Biol. 2023 Nov 29;21:276. doi: 10.1186/s12915-023-01767-z (PMC10688063; doi:10.1186/s12915-023-01767-z)
Supplement: Supplementary file 20 — Additional file 20: Fig. S9. Association between tRNAs and specific AGOs proteins. A. Plot of total tRNAs count (in reads per million) in wild type C. elegans (N2; black diamonds) and mutants lacking specific AGOs proteins (from left to right: ergo1 (blue diamonds), rde1 (yellow diamonds) and wago10 (red diamonds). A very small decrease in wago10 was observed. N = 3 per mutant. B. Plot representing specifically Glutamate tRNA (GluCTC (diamonds) and GluTCC (dots) fragments (in reads per million) in wild type C. elegans (N2; black) and in mutants lacking wago10 (red). N = 5 for N2 with 2 replicates for GluCTC and 3 for GluTCC. N = 6 for wago10 mutant with 3 replicates for both tRNAs. A significant difference in Glutamate tRNA fragments was observed between wild type worms and mutants lacking wago10 (Wilcoxon-test, adjusted p-value = 0.05). Supporting data are available in the excel file: "Additional file 34". [file 12915_2023_1767_MOESM20_ESM.pdf]

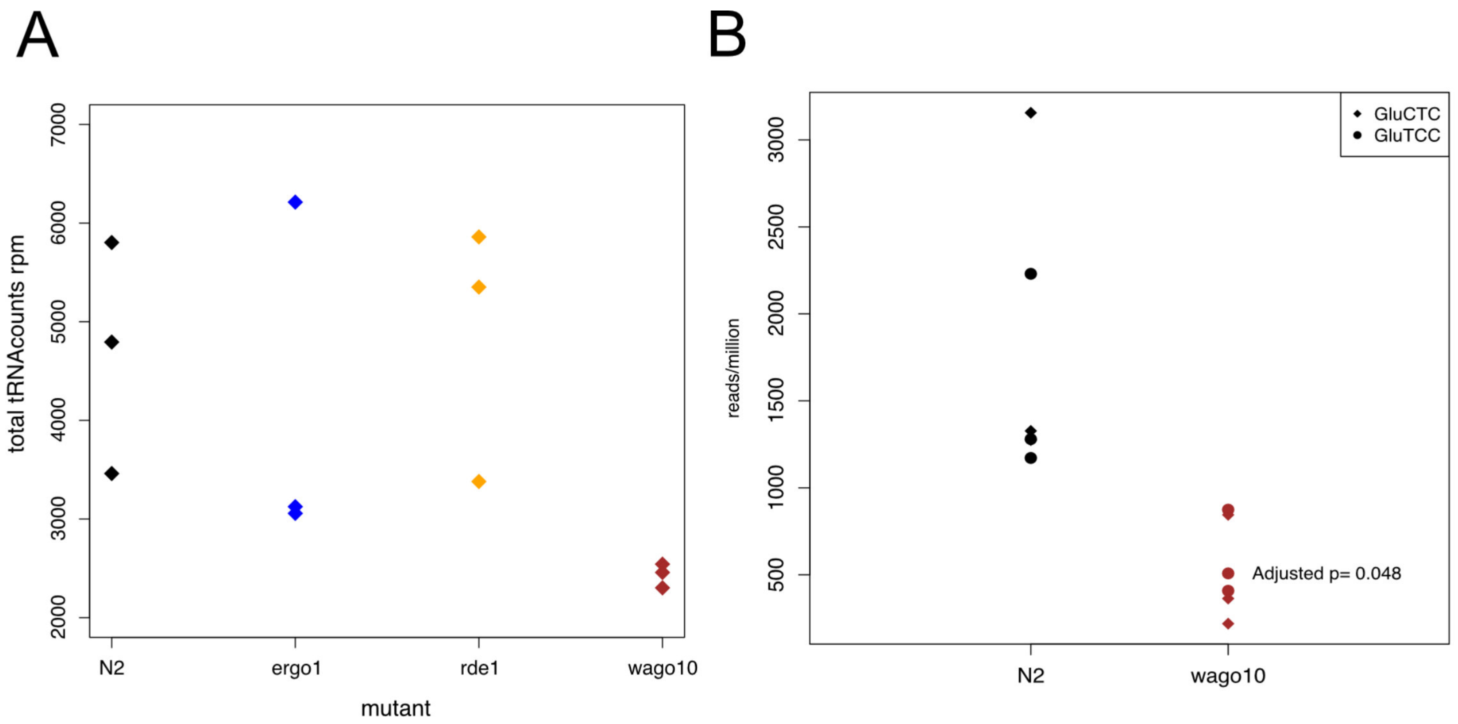

Fig. S9: Association between tRNAs and specific AGOs proteins. A. Plot of total tRNAs count (in reads per million) in wild type *C. elegans* (N2; black diamonds) and mutants lacking specific AGOs proteins (from left to right: ergo1 (blue diamonds), rde1 (yellow diamonds) and wago10 (red diamonds). A very small decrease in wago10 was observed. N = 3 per mutant. B. Plot representing specifically Glutamate tRNA (GluCTC (diamonds) and GluTCC (dots) fragments (in reads per million) in wild type *C. elegans* (N2; black) and in mutants lacking wago10 (red). N = 5 for N2 with 2 replicates for GluCTC and 3 for GluTCC. N = 6 for wago10 mutant with 3 replicates for both tRNAs. A significant difference in Glutamate tRNA fragments was observed between wild type worms and mutants lacking wago10 (Wilcoxon-test, adjusted p-value = 0.05).
Supporting data are available in the excel file: "Additional file 34".

Figure S9
