# Supplementary material for: Cisplatin exposure alters tRNA-derived small RNAs but does not affect epimutations in C. elegans
Source: BMC Biol. 2023 Nov 29;21:276. doi: 10.1186/s12915-023-01767-z (PMC10688063; doi:10.1186/s12915-023-01767-z)
Supplement: Supplementary file 22 — Additional file 22: Fig. S10. Detailed characterisation of tRNAs epimutations. A. Barplot of total number of epimutations for each tRNAs type and in the different conditions: control (blue), LD (green) and HD (red). B. Barplot of the duration of tRNAs epimutations for each kind and according to the exposure condition: control (blue), LD (green) and HD (red). C. Forest plot of Cox Proportional Hazards Model representing the odd of difference in the tRNAs 3’ halves epimutations between the conditions. The x-axis show the chances of an epimutation to disappear in the cisplatin conditions in comparison to control (reference). The p-values were calculated using log rank test. For A, B and C, two lineages for each condition were used as biological replicates. Supporting data can be found in the excel file: "Additional file 35". [file 12915_2023_1767_MOESM22_ESM.pdf]

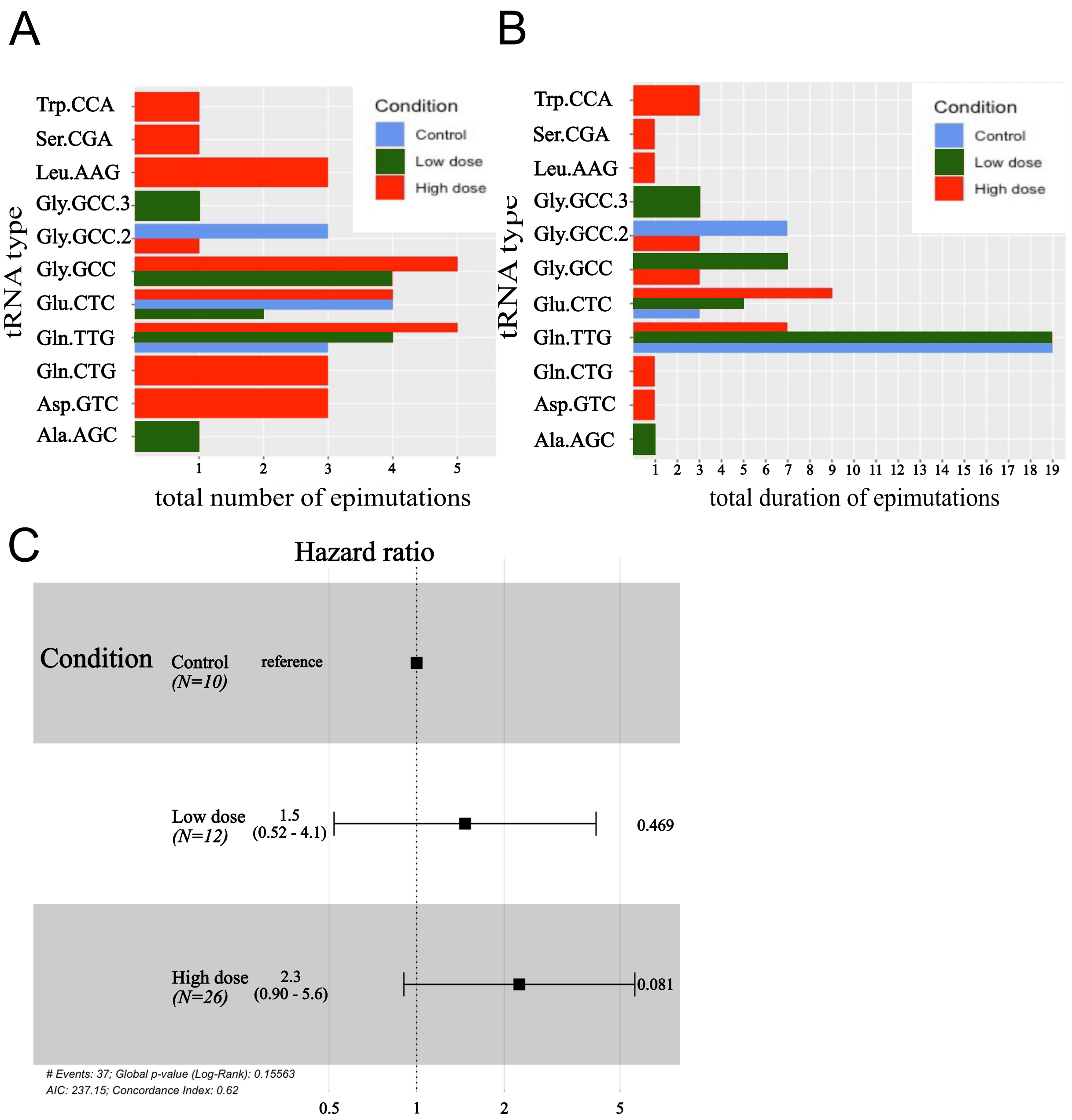

Fig. S10: Detailed characterisation of tRNAs epimutations. A. Barplot of total number of epimutations for each tRNAs type and in the different conditions: control (blue), LD (green) and HD (red). B. Barplot of the duration of tRNAs epimutations for each kind and according to the exposure condition: control (blue), LD (green) and HD (red). C. Forest plot of Cox Proportional Hazards Model representing the odd of difference in the tRNAs 3' halves epimutations between the conditions. The x-axis show the chances of an epimutation to disappear in the cisplatin conditions in comparison to control (reference). The p-values were calculated using log rank test.

For A, B and C, two lineages for each condition were used as biological replicates.

Supporting data can be found in the excel file: "Additional file 35".

Figure S10
